# Explicitly accounting for needle sugar pool size crucial for predicting intra‐seasonal dynamics of needle carbohydrates δ^18^O and δ^13^C

**DOI:** 10.1111/nph.18227

**Published:** 2022-06-24

**Authors:** Kersti Leppä, Yu Tang, Jérôme Ogée, Samuli Launiainen, Ansgar Kahmen, Pasi Kolari, Elina Sahlstedt, Matthias Saurer, Pauliina Schiestl‐Aalto, Katja T. Rinne‐Garmston

**Affiliations:** ^1^ Natural Resources Institute Finland 00790 Helsinki Finland; ^2^ Faculty of Agriculture and Forestry, Institute for Atmospheric and Earth System Research (INAR)/Forest Sciences University of Helsinki 00014 Helsinki Finland; ^3^ UMR ISPA, INRA 33140 Villenave d'Ornon France; ^4^ Department of Environmental Sciences – Botany University of Basel 4056 Basel Switzerland; ^5^ Faculty of Science, Institute for Atmospheric and Earth System Research (INAR)/Physics University of Helsinki 00014 Helsinki Finland; ^6^ Forest Dynamics, Swiss Federal Institute for Forest Snow and Landscape Research (WSL) 8903 Birmensdorf Switzerland

**Keywords:** boreal forest, carbon isotope, dynamic modeling, needle sugar, oxygen isotope, photosynthesis, Scots pine (*Pinus sylvestris*)

## Abstract

- We explore needle sugar isotopic compositions (δ^18^O and δ^13^C) in boreal Scots pine (*Pinus sylvestris*) over two growing seasons.
- A leaf‐level dynamic model driven by environmental conditions and based on current understanding of isotope fractionation processes was built to predict δ^18^O and δ^13^C of two hierarchical needle carbohydrate pools, accounting for the needle sugar pool size and the presence of an invariant pinitol pool.
- Model results agreed well with observed needle water δ^18^O, δ^18^O and δ^13^C of needle water‐soluble carbohydrates (sugars + pinitol), and needle sugar δ^13^C (*R* ^2^ = 0.95, 0.84, 0.60, 0.73, respectively). Relative humidity (RH) and intercellular to ambient CO_2_ concentration ratio (*C*_i_/*C*_a_) were the dominant drivers of δ^18^O and δ^13^C variability, respectively. However, the variability of needle sugar δ^18^O and δ^13^C was reduced on diel and intra‐seasonal timescales, compared to predictions based on instantaneous RH and *C*_i_/*C*_a_, due to the large needle sugar pool, which caused the signal formation period to vary seasonally from 2 d to more than 5 d. Furthermore, accounting for a temperature‐sensitive biochemical ^18^O‐fractionation factor and mesophyll resistance in ^13^C‐discrimination were critical.
- Interpreting leaf‐level isotopic signals requires understanding on time integration caused by mixing in the needle sugar pool.

## Abstract 2

See also the Commentary on this article by Ubierna *et al*., **236**: 2003–2008.

## Introduction

Stable carbon and oxygen isotope compositions in tree rings (δ^13^C and δ^18^O, respectively) provide records of past environmental and tree physiological signals (McCarroll & Loader, 2004; Battipaglia *et al*., 2013). Tree ring δ^13^C and δ^18^O records are foreseen as powerful tools in advancing our understanding on the response of forests to changing climate and increasing atmospheric CO_2_ concentration (Gessler *et al*., 2014). The formation of tree ring δ^13^C and δ^18^O signals in boreal coniferous species is of special interest, because these sensitive ecosystems play a critical role in the carbon cycle of our planet (Snyder *et al*., 2004) and are now undergoing climatic warming at a significantly faster rate compared to the global average (IPCC, 2021). To this end, we need to understand how isotopic signals are formed in the leaves, where most of the interaction with local environmental conditions occurs. Even though, this is a vastly studied field, intra‐seasonal studies in boreal forest are scarce.

Newly assimilated photosynthates are products of atmospheric CO_2_ and leaf water, thus reflecting δ^13^C of atmospheric CO_2_ strongly modified by leaf biochemistry (Farquhar *et al*., 1982) and δ^18^O of source water that is further ^18^O‐enriched in leaves during evaporation (Farquhar & Lloyd, 1993). ^18^O‐enrichment due to evaporation is linked to the variation of relative humidity (RH) as defined by the model of Craig & Gordon (1965), developed for open water bodies and adapted later to leaves (Dongmann *et al*., 1974). The Craig–Gordon model is, however, prone to overestimate ^18^O‐enrichment (Cernusak *et al*., 2016), which has led to the introduction of the Péclet effect (Farquhar & Lloyd, 1993; Barbour *et al*., 2000) and the two‐pool concept (Leaney *et al*., 1985; Roden *et al*., 2015). The δ^18^O of leaf photosynthates is expected to reflect the δ^18^O of leaf water with an offset of 27‰ (Barbour *et al*., 2000; Cernusak *et al*., 2003). Although the offset 27‰ is commonly assumed constant, Sternberg & Ellsworth (2011) reported it to be inversely related to temperature, with a particularly strong incline below 20°C. Recently, Hirl *et al*. (2021) acknowledged the need to account for this temperature dependence, when modeling the seasonal variation of cellulose δ^18^O in a grassland ecosystem, underlining the power of modeling in testing our current theoretical understanding. In cooler boreal conditions, such temperature dependence is potentially of even higher importance.

Regarding δ^13^C, the pathway to primary photosynthates in C_3_ plants can be divided into fractionation by diffusion from air to chloroplast through stomata and mesophyll, and biochemical fractionation, that is, carboxylation, mitochondrial respiration and photorespiration (Farquhar *et al*., 1982; Cernusak *et al*., 2013). Recent studies have emphasized the importance of dynamic mesophyll conductance on δ^13^C of assimilates (Stangl *et al*., 2019; Schiestl‐Aalto *et al*., 2021). Mechanistic modeling studies have further shown that tree ring δ^13^C signals are highly sensitive to isotopic fractionation during both photosynthesis and respiration (Ogée *et al*., 2009; Eglin *et al*., 2010) and that the sugars used for leaf respiration may have a δ^13^C value that differs from current assimilates (Wingate *et al*., 2007). These findings together with the fact that the fractionation factors of these processes are still poorly constrained *in vivo* (Barbour & Song, 2014), complicate modeling δ^13^C signals in plants.

Interpreting and predicting isotopic signals in leaves may be further complicated in field conditions with strong variability in environmental conditions over sub‐daily to seasonal timescales. For example, leaf water δ^18^O sampled on sub‐daily timescales in varying ambient conditions has been shown to deviate significantly from values predicted by a steady‐state model (Farquhar & Cernusak, 2005; Gessler *et al*., 2013). Similar nonsteady‐state effects are expected, when predicting the isotopic composition of leaf sugars as the sugar pool does not represent only the current assimilates (Barnard *et al*., 2007; Gessler *et al*., 2013). There are dynamic models describing intra‐seasonal variation in δ^13^C and δ^18^O in tree ring cellulose (e.g. Ogée *et al*., 2009; Eglin *et al*., 2010), but similar descriptions for leaf sugar pools are scarce. These earlier studies face the difficulty of differentiating between fractionation processes in the leaves and during the pathway to tree rings, which stresses the need for leaf‐level mechanistic models.

Measurements of the isotopic composition of leaf sugars is challenging in many respects. Primarily, studies often analyze only mixtures of compounds (e.g. total leaf organic matter, water‐soluble organic matter, or purified water‐soluble extracts), which tends to reduce the temporal variability of the isotopic signal of interest, as compounds within the mixture can have different live‐spans in the leaf and differ in their isotope value due to fractionations during secondary metabolism (Barnard *et al*., 2007; Offermann *et al*., 2011; Gessler *et al*., 2013; Rinne *et al*., 2015; Lehmann *et al*., 2020). Purified water extracts (hereafter water‐soluble carbohydrates, WSC) are expected to consist mainly of sugars (Richter *et al*., 2009; Rinne *et al*., 2015) and have therefore potentially more variable isotopic signal than total organic matter. However, if sugar alcohols (e.g. pinitol/myo‐inositol, hereafter referred to as pinitol; see Rinne *et al*., 2012) are present in high concentrations, they have been shown to reduce the variability of the δ^13^C signal of WSC (Richter *et al*., 2009; Rinne *et al*., 2015). Permanent high fraction of pinitol throughout the growing season is typical for conifer needles in high‐latitude and high‐elevation conditions (Lipavská *et al*., 2000; Streit *et al*., 2013; Rinne *et al*., 2015). To address this issue, compound‐specific isotope analysis (CSIA) of δ^13^C provides means to retrieve the isotopic signal of e.g. sucrose, the main transport sugar, from the bulk sample (Rinne *et al*., 2012). For δ^18^O, the analytical process is more laborious and only a few studies have reported CSIA results for a limited number of samples (Lehmann *et al*., 2016, 2017). Intra‐seasonal CSIA of δ^18^O are therefore still out of reach for modeling purposes. However, making use of the CSIA results of δ^13^C, compound concentrations, and concurrently analyzed δ^13^C and δ^18^O of WSC, mechanistic models can attempt to answer how variable δ^18^O is between various compounds.

This study explores the formation of needle sugar isotopic signals in boreal Scots pine over two growing seasons in southern Finland. We concurrently present observations and environmentally driven dynamic modeling of the intra‐seasonal variation of δ^18^O and δ^13^C of needle WSC. Our isotopic data consists of δ^18^O in water pools (e.g. twig and needle), δ^18^O and δ^13^C analyzed from WSC as bulk, and δ^13^C results from CSIA. Combining mechanistic modeling and data, we aim to address the following research questions:

- Which fractionation and mixing processes are important for capturing intra‐seasonal variation of needle sugar δ^18^O and δ^13^C?
- What is the role of the sugar pool size in predicting needle sugar δ^18^O and δ^13^C?
- What implications do needle sugar pool size and the composition of bulk samples have on interpreting environmental/physiological signals from needle δ^18^O and δ^13^C?

## Model description

The built model describes the dynamics of δ^18^O and δ^13^C in two hierarchical needle carbohydrate pools in response to half‐hourly environmental conditions (air temperature, photosynthetic active radiation (PAR), vapor pressure, atmospheric CO_2_ concentration, and soil moisture) and isotopic input data (δ^18^O of water vapor and source water, and δ^13^C of atmospheric CO_2_). The two carbohydrate pools described in the model are needle: (1) sugars; and (2) WSC, which in addition to sugars contain pinitol. The model builds on a photosynthesis model solving leaf net CO_2_ exchange, including carboxylation, photorespiration, and mitochondrial respiration (see Supporting Information Methods S1; Table S1). Doing so it accounts for stomatal control and mesophyll resistance, but neglects leaf energy balance assuming the leaf is at air temperature (a fair assumption, given the small size of Scots pine needles that are well‐coupled to the atmosphere; Launiainen *et al*., 2016; Kim *et al*., 2018). Theory‐based isotopic fractionation of oxygen and carbon in needles and their accumulation in the needle sugar and WSC pools are then solved as described in the following section. Model equations are expressed in terms of isotopic ratios (*R*), which can be converted to ‘delta’ notations:

(Eqn 1) $$\delta =\left(\frac{R}{R_{\mathrm{std}}}-1\right)$$

where *R*_std_ is the isotope ratio of an international standard; Vienna Standard Mean Ocean Water (VSMOW) for ^18^O/^16^O and Vienna Peedee belemnite (VPDB) for ^13^C/^12^C.

### Formation of needle sugar δ^18^O

In steady‐state conditions, the isotopic ratio of oxygen at evaporative sites is given by Flanagan *et al*. (1991)

(Eqn 2) $$R_{e,\mathrm{ss}}=\alpha^{+}\left(\alpha_{k}\frac{w_{i}-w_{a}}{w_{i}}R_{s}+\frac{w_{a}}{w_{i}}R_{v}\right)$$

where *R*_s_ and *R*_v_ are the isotopic ratios of oxygen in source water and atmospheric water vapor, respectively; *α* ^+^ (= 1 + *ε* ^+^) is the temperature‐dependent equilibrium fractionation during vaporization (Majoube, 1971); *w*_a_ and *w*_i_ (in mol mol^−1^) are the mole fractions of water vapor in the atmosphere and inside the leaf, respectively; and *α*_k_ is the kinetic isotope fractionation during water vapor diffusion through stomata and leaf boundary layer:

(Eqn 3) $$\alpha_{k}=1+\frac{g_{b}\varepsilon_{\mathrm{ks}}+g_{s}\varepsilon_{\mathrm{kb}}}{g_{b}+g_{s}}$$

where *g*_s_ and *g*_b_ (in mol m^−2^ s^−1^) are stomatal and boundary layer conductances for CO_2_ (assuming both scale for water vapor by 1.6); and *ε*_ks_ and *ε*_kb_ are the fractionation factors associated with the diffusion through stomata and the boundary layer, respectively (Table 1).

**Table 1:** Parameters for isotopic modeling.

Parameter^a^	Description	Value	Source
$a_{b}$ (−)	Fractionation during diffusion of CO_2_ through the boundary layer	2.9‰	Farquhar (1983)
$a_{s}$ (−)	Fractionation during diffusion of CO_2_ through stomata	4.4‰	O'Leary (1981)
$a_{m}$ (−)	Fractionation during transfer of CO_2_ through mesophyll	1.8‰	O'Leary (1984)
*b* (−)	Fractionation during caboxylation	29‰	Roeske & O'Leary (1984)
*f* (−)	Fractionation during photorespiration	8‰	Ghashghaie *et al*. (2003)
*e* (−)	Fractionation during mitochondrial respiration	–6‰	Ghashghaie *et al*. (2003)
δ^13^C_pin_ (−)	Pinitol δ^13^C in needle	−30.5‰	Measurements, see Fig. S2(d)
$\varepsilon^{+}$ (−)	Equilibrium fractionation during vaporization	–	Majoube (1971)
$\varepsilon_{\mathrm{kb}}$ (−)	Fractionation during diffusion of water vapor through boundary layer	19‰	Merlivat (1978)
$\varepsilon_{\mathrm{ks}}$ (−)	Fractionation during diffusion of water vapor through stomata	28‰	Merlivat (1978)
*f*_1_ (−)^b^	Ratio of enriched to total needle water	0.93	Calibrated
*L* (m)^c^	Leaf mesophyll effective mixing length	0.03	Calibrated
*W* (mol m^−2^)	Leaf mesophyll water volume	5.6	Measurements, see Fig. S6
$\varepsilon_{\mathrm{wc}}$ (−)	Biochemical fractionation factor	27‰ $\varepsilon_{\mathrm{wc}}\left(T\right)$	Sternberg *et al*. (1986) fig. 1 in Sternberg & Ellsworth (2011)
δ^18^O_pin_ (−)	Pinitol δ^18^O in needle	25‰	Calibrated
*S*_sug_ (μmol of C m^−2^)	Concentration of needle sugar	1.96 × 10^5^	Measurements, see Fig. S5
*S*_pin_ (μmol of C m^−2^)	Concentration of needle bulk sugars	0.7 *S*_sug_	Measurements, see Fig. S5(b)

^a^ Parameter units are given in parenthesis, where ‘–’ stand for unitless and all area‐based units refer to all‐sided leaf area.

^b^ Two‐pool approach.

^c^ Péclet approach.

Average mesophyll water (*R*_lw,ss_) is less ^18^O‐enriched compared to the sites of evaporation, which at steady state is commonly formulated as a reduction factor (*f*_1_) above source water:

(Eqn 4) $$R_{\mathrm{lw},\mathrm{ss}}=f_{1}\left(R_{e,\mathrm{ss}}-R_{s}\right)+R_{s}$$

There are two alternative concepts for defining *f*_1_: (1) the two‐pool model, where *f*_1_ is a constant (Leaney *et al*., 1985); and (2) the Péclet model, which relates *f*_1_ to transpiration (Farquhar & Lloyd, 1993). In the latter $f_{1}=\left(1-e^{-℘}\right)/℘$, where $℘=\mathrm{EL}/\left(\mathrm{CD}\right)$; *L* (in meters) is the effective mixing length, *E* (in mol m^−2^ s^−1^) the transpiration rate, *C* (55.5 × 10^3^ mol m^−3^) the molar density of liquid water, and *D* (2.66 × 10^−9^ m^2^ s^−1^) the diffusivity of H_2_^18^O in liquid water.

At sub‐daily timescales and at times of low transpiration, the steady‐state assumption may be violated because of the slow turn over rate of leaf water (Farquhar & Cernusak, 2005; Gessler *et al*., 2013). The formulation for the isotopic ratio of oxygen in leaf water under nonsteady state (*R*_lw_) is given by (Farquhar & Cernusak, 2005)

(Eqn 5) $$\frac{{\mathrm{dWR}}_{\mathrm{lw}}}{\mathrm{dt}}=\frac{{\mathrm{Ew}}_{i}f_{1}}{\alpha^{+}\alpha_{k}\left(w_{i}-w_{a}\right)}\left(R_{\mathrm{lw},\mathrm{ss}}-R_{\mathrm{lw}}\right)$$

where *W* (in mol m^−2^) is the leaf water content.

New assimilates are generally assumed to be in oxygen isotopic equilibrium with bulk leaf water (Barbour *et al*., 2000): $R_{\text{assimilates}}=\alpha_{\mathrm{wc}}R_{\mathrm{lw}}$, where *α*_wc_ (= 1 + *ε*_wc_) is the biochemical fractionation associated with oxygen isotope exchange between carbonyl oxygen and water (Sternberg *et al*., 1986; Sternberg & Ellsworth, 2011). The signal of new assimilates is then carried to the needle sugar pool following Eqn 6, assuming that the needle sugar pool is well‐mixed and forms the substrate for mitochondrial respiration (Wingate *et al*., 2007; Ogée *et al*., 2009).

(Eqn 6) $$\frac{d\left(S_{\mathrm{sug}}R_{\mathrm{sug}}\right)}{\mathrm{dt}}=\left(A_{n}+r_{d}\right)R_{\text{assimilates}}-r_{d}R_{\mathrm{sug}}-{\mathrm{qR}}_{\mathrm{sug}}$$

where *R*_sug_ is the isotopic ratio in the needle sugar pool, *S*_sug_ (in μmol of C m^−2^) the concentration of needle sugar, *A*_n_ net CO_2_ exchange, *r*_d_ mitochondrial respiration, and *q* the discharge of sugars from the needle into the phloem (all in μmol m^−2^ s^−1^). Finally, the isotopic ratio of needle WSC is computed as

(Eqn 7) $$R_{\mathrm{wsc}}=\frac{S_{\mathrm{sug}}R_{\mathrm{sug}}+S_{\mathrm{pin}}R_{\mathrm{pin}}}{S_{\mathrm{sug}}+S_{\mathrm{pin}}}$$

where *R*_pin_ and *S*_pin_ (in μmol of C m^−2^) are the isotopic ratio and concentration of pinitol in the needle, respectively.

### Formation of needle sugar δ^13^C

The model for ^13^C‐discrimination of net CO_2_ exchange (${}^{13}\Delta$, Eqn 8) was adopted from Wingate *et al*. (2007) (see also Methods S2). They modify the classical equation by Farquhar *et al*. (1982) to account for the fact that the δ^13^C composition of the substrate used for mitochondrial respiration may differ from that of current assimilates. Here we assume that substrate is the needle sugar pool. Unlike the formulation in Farquhar *et al*. (1982) that requires photosynthesis to model the δ^13^C of the respiratory substrates, this formulation is also valid in absence of photosynthesis (Wingate *et al*., 2007); during nighttime (*k* = 0), it simply reduces to the ^13^C‐discrimination of dark respiration.

(Eqn 8) $${}^{13}\Delta =\frac{{\mathrm{kC}}_{a}}{{\mathrm{kC}}_{a}-r_{d}}\left(a_{b}\frac{C_{a}-C_{s}}{C_{a}}+a_{s}\frac{C_{s}-C_{i}}{C_{a}}+a_{m}\frac{C_{i}-C_{c}}{C_{a}}+b\frac{C_{c}}{C_{a}}-f\frac{\Gamma_{*}}{C_{a}}\right)-\frac{r_{d}}{{\mathrm{kC}}_{a}-r_{d}}\left(\frac{R_{a}}{R_{\mathrm{sug}}}\left(1+e\right)-1\right)$$

where *C*_a_, *C*_s_, *C*_i_ and *C*_c_ (in μmol mol^−1^) are CO_2_ mole fractions in the atmosphere, at the leaf surface, in the intercellular spaces and in the chloroplasts; *R*_a_ and *R*_sug_ are isotopic ratios of CO_2_ in ambient air and in the sugar pool; *a*_b_, *a*_s_, *a*_m_, *b*, *f* and *e* are fractionation factors associated with diffusion through the boundary layer, diffusion through stomata, transfer through mesophyll, carboxylation, photorespiration, and mitochondrial respiration, respectively (Table 1); Γ_*_ (in μmol mol^−1^) is the CO_2_ compensation point in the absence of mitochondrial respiration (Bernacchi *et al*., 2001) and; *k* (= (*A*_n_ + *r*_d_)/(*C*_c_ – Γ_*_)) is the carboxylation efficiency. Eqn 8 ignores ternary effects as they are also ignored in modeling shoot gas exchange (Methods S1). Such approach is common for mechanistic modeling studies simulating both gas exchange and ^13^C‐discrimination (Ogée *et al*., 2009; Schiestl‐Aalto *et al*., 2021). According to Farquhar & Cernusak (2012) ignoring or accounting for ternary effects consistently in both gas exchange and discrimination calculations lead to almost equivalent results, whereas their inconsistent use in either gas exchange or discrimination calculations produce misleading results. Eqn 8 further assumes there is effectively only one carbon pool, where carbon compounds left behind by respiratory processes mix with the pool of respiratory substrate and carbon in the Calvin–Benson–Bassham (CBB) cycle. Busch *et al*. (2020) recently suggested an alternative model where respiration is isotopically disconnected and metabolites are not fed back into the CBB cycle, but this possibility was not included in our analysis.

The isotopic ratio of net CO_2_ exchange is *R*_a_/(1 + ^13^Δ), thus the isotopic signal of carbon in the needle sugar pool is solved from:

(Eqn 9) $$\frac{d\left(S_{\mathrm{sug}}R_{\mathrm{sug}}\right)}{\mathrm{dt}}=A_{n}\frac{R_{a}}{\left(1+{\;}^{13}\Delta \right)}-{\mathrm{qR}}_{\mathrm{sug}}$$

The δ^13^C of the needle WSC pool is computed as for oxygen (Eqn 7). The right‐hand side of Eqn 9 is equivalent to $\left(A_{n}+r_{d}\right)R_{\text{assimilates}}-r_{d}\left(1-e\right)R_{\mathrm{sug}}-{\mathrm{qR}}_{\mathrm{sug}}$.

## Materials and Methods

### Study site and measurements

#### Site description

The study site is the Station for Measuring Forest Ecosystem‐Atmosphere Relations (SMEAR II) in Hyytiälä, southern Finland (61°51′N, 24°17′E; Hari & Kulmala, 2005). The site is a managed boreal forest on a shallow mineral soil, with an overstorey dominated by 60‐yr‐old Scots pines (*Pinus sylvestris* L.). The long‐term (1981–2010) mean annual temperature and precipitation is 3.5°C and 711 mm, respectively (Pirinen *et al*., 2012). Snow typically covers the ground from December to April.

#### Shoot gas exchange

Shoot gas exchange measurements were performed with an automated chamber system (Aalto *et al*., 2014) consisting of a chamber, sample tubing and a gas analyzer. The box shaped shoot chamber (2.1 dm^3^) made of acrylic plastic and inner surfaces coated with fluorinated ethylene propylene (FEP) film was installed in the uppermost canopy. The chamber enclosed a 1‐yr‐old (in 2018) shoot in horizontal position. The inserted shoot was debudded before the chamber installation to prevent new growth. The chamber was open most of the time exposing the shoots to ambient conditions and closed intermittently for 1 min 50–90 times d^–1^. Sample air was drawn from the chamber along polytetrafluoroethylene tubes (internal diameter 4 mm, length 73 m) to a gas analyzer (LI‐840; Li‐Cor, Lincoln, NE, USA) and compensated by ambient air leaking freely into the chamber. Fluxes of CO_2_ and water were determined by fitting a nonlinear equation to concentration records during the first 5–35 s of chamber closure (Kolari *et al*., 2012). Water fluxes were rejected when RH was > 85% due to considerable adsorption of water on the chamber walls. Fluxes were calculated for all‐sided needle area (Kolari *et al*., 2007) and re‐sampled to half‐hourly time series. Based on all‐sided needle area measurements the specific leaf area was 0.010 m^2^ g^−1^. Resulting data coverage for fluxes of CO_2_ and water were 98% and 63%, respectively, for mid‐April to mid‐October in 2018–2019.

#### Sampling and isotopic analysis of water

Samples of 1‐yr‐old needle (1N) water, twig water, soil water and atmospheric water vapor were collected every 3–5 wk during May–October in 2018 and 2019, except for 1N water sampled more frequently in 2019 (14 times + diurnal course on 23 May). Furthermore, 1N and twig samples were collected from five mature Scots pine trees between midday and 15:00 h (utc + 2) from a sun‐exposed position in canopy top. For each tree, 1N samples and the linked twigs with barks peeled off were collected into separate 12 ml exetainer vials (Labco, Lampeter, UK). Soil samples were cored using a foot‐step soil probe from three spots close to the sampled trees. Subsamples were taken from 2, 10 and 18 cm depths, and placed into individual exetainers. Water vapor was collected at 18 m height by pumping air for 2 to 3 h through a hose into a glass U‐tube, which was immersed in a mixture of dry ice and ethanol. Additionally, monthly precipitation samples were collected from May 2018–December 2019 using an evaporation‐free rainwater collector (Gröning *et al*., 2012). All collected samples were placed in a cool box with ice blocks immediately in the field and stored at −20°C.

Water vapor and precipitation (May–November 2018) samples were melted and transferred into 2 ml vials and analyzed for δ^18^O at the Stable Isotope Laboratory of Luke (SILL, Helsinki, Finland) by HT‐EA‐IRMS, that is, high temperature (HT) elemental analyzer (Sercon Ltd, Crewe, UK) connected to isotope ratio mass spectrometry (20–22 IRMS; Sercon). The results were calibrated against two in‐house reference waters. Water from plant and soil samples was cryogenically extracted by vacuum distillation (West *et al*., 2006). The δ^18^O of plant and soil extracted water of samples from 2018 were determined at the Stable Isotope Research Laboratory of WSL (Birmensdorf, Switzerland) (Lehmann *et al*., 2020), while corresponding samples from 2019 and precipitation from December 2018 to December 2019 were analyzed at the Stable Isotope Laboratory of University of Basel, Switzerland. For all samples, measurement precision derived from repeated measurements and a quality control water sample was 0.3‰ or better.

#### Sampling and isotopic analysis of water‐soluble needle carbohydrates

Current‐year needles (0N) and/or 1N were collected *c*. 20 times during May to October in 2018 and 2019 (+ diurnal course on 25 July 2019), from five trees during same time and canopy position as for water sampling. Samples were placed in a cool box with ice blocks immediately after collection and microwaved at 600 W for 1 min within 2 h to stop enzymatic and metabolic activities (Wanek *et al*., 2001). Samples were subsequently dried at 60°C for 24 h and homogenized into a fine powder by a ceramic‐ball mill using FastPrep‐24TM. Extraction and purification of WSC were performed according to Wanek *et al*. (2001) and Rinne *et al*. (2012). Shortly, the supernatant from the water extraction at 85°C was separated and purified using three types of sample treatment cartridges, which removed amino acids, organic acids and phenolic compounds. The purified samples were freeze‐dried, dissolved in Milli‐Q water, filtered through a 0.45 μm syringe filter (Acrodisc; Pall Corp., Port Washington, NY, USA) and stored at −20°C.

Bulk isotope analysis of WSC was performed at SILL. Before analysis, aliquots of solubilized extracted WSC were pipetted into individual tin and/or silver capsules (IVA Analysentechnik, Meerbusch, Germany), lyophilized and wrapped. The δ^13^C values were determined using EA‐IRMS (samples of 2018) or concurrently with δ^18^O analysis by HT‐EA‐IRMS using the dual isotope method (samples of 2019; Woodley *et al*., 2012). The EA‐IRMS results for δ^13^C were calibrated against IAEA‐C7 (−32.15‰), IAEA‐CH3 (−24.72‰) and an in‐house sucrose (−12.22‰; Sigma‐Aldrich) reference materials. The dual isotope measurements (δ^18^O and δ^13^C) were calibrated against IAEA‐601 (23.14‰ and −28.81‰), in‐house sucrose (36.62‰ and −12.22‰) and lactose (21.05‰ and −24.66‰) standards (Sigma‐Aldrich). Additionally, a subset of the samples were analyzed for δ^13^C using both the EA and the HT‐EA method to calibrate the HT‐EA results for δ^13^C (Woodley *et al*., 2012). Analytical precision of the measurements was 0.2‰ or better for δ^13^C and δ^18^O, determined from repeated measurements of a quality control standard.

Compound‐specific isotope analysis of δ^13^C was performed for the WSC samples of 2018 only, as analysis of samples of 2019 were delayed due to instrumental problems. CSIA was done at WSL using a Delta V Advantage IRMS (Thermo Fisher Scientific, Waltham, MA, USA) coupled to a high‐performance liquid chromatography (HPLC) system with a Finnigan LC Isolink interface (Thermo Fisher Scientific) (Rinne *et al*., 2012). The δ^13^C values were determined for the four detected sugars or sugar‐like compounds: sucrose, glucose, fructose and pinitol. External compound‐matched standard solutions with comparable concentration (20–180 ng C μl^−1^) and δ^13^C values, as determined by EA‐IRMS, were analyzed between every 10 samples to correct the CSIA results (Rinne *et al*., 2012). The measurement precision (standard deviation, SD) of sucrose, glucose, fructose and pinitol standards were 0.44‰, 0.57‰, 0.88‰, and 0.38‰, respectively.

The concentration of each four individual compounds from HPLC‐IRMS were calculated using its peak area, and the linear regression between the carbon content and peak area of the compound‐matched standard (Rinne *et al*., 2012). The concentration of bulk WSC was calculated from the ratio of the sample weight in the tin/silver capsule to the sample weight used for the hot water extraction.

Based on CSIA, needle sugars comprised mainly of sucrose, while the total contribution of glucose and fructose was on average 25%. The concentration‐weighted average δ^13^C of the sugars was highly correlated with δ^13^C of sucrose (*r* > 0.95; Fig. S1), and hence δ^13^C of sucrose is used to indicate the δ^13^C of the total needle sugars hereafter.

The δ^18^O and δ^13^C of bulk WSC, and δ^13^C of sucrose and pinitol were each combined over the two needle generations (0N and 1N) to form time series covering the whole growing season (Fig. S2). The δ^18^O of bulk WSC and δ^13^C of sucrose were similar for 0N and 1N and the combined dataset was obtained as their average (Fig. S2a,c). Bulk WSC in 2018 and pinitol showed a difference in level of δ^13^C between 0N and 1N, which was first corrected for before averaging over the two data series (Fig. S2b,d).

### Model runs, parameterization and evaluation

The model was ran for the two growing seasons, 2018–2019, using half‐hourly meteorological data measured above the canopy. Additionally, soil moisture from *c*. 5 cm depth in mineral soil was used as proxy for water availability limiting photosynthesis during dry conditions (Eqn S10 in Methods S1; Launiainen *et al*., 2022). Isotopic input data was not measured at the site at the resolution needed for the modeling. The δ^18^O of water vapor (six‐hourly) was obtained from the isotope‐enabled, nudged atmospheric general circulation model IsoGSM (Yoshimura *et al*., 2011, 2008), which corresponded reasonably well to the limited number of observations (Fig. S3). The δ^18^O of source water was modeled at daily resolution based on monthly precipitation δ^18^O, the amount of precipitation, soil moisture and eddy covariance‐based evapotranspiration, resulting in a good fit with the observed twig water δ^18^O (see Methods S3; Fig. S4). Lastly, δ^13^C of atmospheric CO_2_ was available at weekly resolution from Pallas‐Sammaltunturi GAW‐station (White *et al*., 2015).

Initially, the model results related to leaf gas exchange (*A*_n_, *E* and *C*_i_/*C*_a_) were evaluated against shoot chamber measurements. Thereafter, the results of the isotopic model were compared against measured δ^18^O of leaf water, δ^18^O and δ^13^C of needle WSC, and δ^13^C of needle sugars. The model fit was evaluated using mean absolute error (MAE) and *R* ^2^.

The parameters applied in the isotopic modeling are listed in Table 1. The measured WSC concentrations indicated little variability during the growing seasons (Fig. S5a). The concentrations fluctuated *c*. 95 mg g^−1^, which corresponds to 3.33 × 10^5^ μmol of C m^−2^, when using the measured specific leaf area (0.010 m^2^ g^−1^) and the molecular mass of carbon in sucrose 28.5 g (mol C)^−1^ (similar to 27.7 and 30 g (mol C)^−1^ for pinitol and glucose/fructose, respectively). The ratio of pinitol to total sugar concentration was also rather invariant during the measurement period, especially after the beginning of July (Fig. S5b). The ratio was *c.* 0.7, meaning that sugars accounted for 59% of WSC and pinitol for 41%. Thus, *S*_sug_ was defined as a constant (Table 1) and hence the discharge from the sugar pool in Eqns 6 and 9 was set equal to net CO_2_ exchange (*q* = *A*_n_). Lastly, pinitol δ^13^C was set to −30.5‰ based on CSIA results (Fig. S2d), whereas pinitol δ^18^O was calibrated in the absence of CSIA results for oxygen.

For both oxygen and carbon isotopic models, we applied a selection of model variants to understand the role of different processes in capturing intra‐seasonal variation of needle sugar δ^13^C and δ^18^O. For oxygen, the model variants for needle water δ^18^O included the Craig–Gordon model (Eqn 2), the two‐pool model (Eqn 4 with constant *f*_1_) and the Péclet model (Eqn 4 with transpiration‐dependent *f*_1_). Each of these models were run both in steady and nonsteady state. Furthermore, to predict needle WSC δ^18^O, we tested the model with a constant and a temperature‐dependent *ε*_wc_ (Table 1); and by defining the size of the sugar pool corresponding to the measurements (Table 1) or as a 20‐fold smaller value. The latter resulted in δ^18^O of leaf water with an offset of *ε*_wc_ (or δ^13^C of net CO_2_ exchange) to be approximately equal to the isotopic values of the needle sugar pool (i.e. all sugars in needle represent current assimilates).

For carbon, we tested how much neglecting mesophyll resistance affects the model results by assuming *C*_c_ = *C*_i_. The importance of photorespiration and mitochondrial respiration were tested by setting *f* and *r*_d_ to zero, respectively. With *f* = *r*_d_ = 0, Eqn 8 reduces to its most simple formulation. In addition to *b* = 29‰, this most simple formulation was ran with *b* = 27‰ and *C*_c_ = *C*_i_, where the lower *b* implicitly accounts for all isotopic effects that happen during photosynthetic discrimination including the contribution of mesophyll resistance (Farquhar *et al*., 1982; Ubierna & Farquhar, 2014). With *r*_d_ = 0, Eqn 8 reduces to the commonly applied formulation that only accounts for diffusion, carboxylation and photorespitation (e.g. Seibt *et al*., 2008), whereas setting *e* = 0 would still consider the release of respired CO_2_ into intercellular spaces (Wingate *et al*., 2007). Finally, as for oxygen, the effect of the size of the sugar pool was investigated.

### Evaluating formation period and environmental/physiological signals

For model results and observations, we examined the relationships between isotopic signals and environmental/physiological variables, that is, needle sugar δ^18^O against RH and δ^13^C against *C*_i_/*C*_a_. Pearson's correlation coefficient (*r*) was used to quantify the strength of the linear relationship.

To account for the integration over time in the needle sugar pool, we calculated a weighted mean of past RH and *C*_i_/*C*_a_ based on the same assumptions as implemented in the model (i.e. well‐mixed sugar pool of constant size). The implicit solution of Eqn 6 (or Eqn 9 when neglecting *e*) defines the sugar pool signal at time *t* as:

(Eqn 10) $$R_{\mathrm{sug}}^{t}=\alpha^{t}R_{\text{assimilates}}^{t}+\left(1-\alpha^{t}\right)R_{\mathrm{sug}}^{t-1}$$

where $\alpha =\left(A_{n}+r_{d}\right)/\left(S_{\mathrm{sug}}/\Delta t+A_{n}+r_{d}\right)$ and Δ*t* (in seconds) is the time interval between *t* − 1 and *t*. Applying Eqn 10 recursively the sugar pool signal can be written as a weighed mean of past time instances $R_{\text{assimilates}}$.

(Eqn 11) $$R_{\mathrm{sug}}^{t}=\sum_{n=0}^{\tau }\left(w_{n}R_{\text{assimilates}}^{t-n}\right)/\sum_{n=0}^{\tau }w_{n}$$

where *τ* is the number of time steps to consider (here cut off at $\sum_{n=0}^{\tau }w_{n}\approx 0.95$) and *w*_n_ is the weight of the signal at time *t* – *n* expressed as:

(Eqn 12) $$w_{n}=\alpha^{t-n}\times \prod_{i=0}^{n-1}\left(1-\alpha^{t-i}\right)$$

Eqn 11 was used to calculate a weighted RH and *C*_i_/*C*_a_ by replacing *R* by RH and *C*_i_/*C*_a_, respectively. Furthermore, the length of the signal formation period (*τ*Δ*t*) over the two growing seasons was examined. For step‐by‐step derivation of Eqns (Eqn 10), (Eqn 11), (Eqn 12) see Methods S4.

## Results

### Environmental conditions and shoot gas exchange

**Fig. 1:**
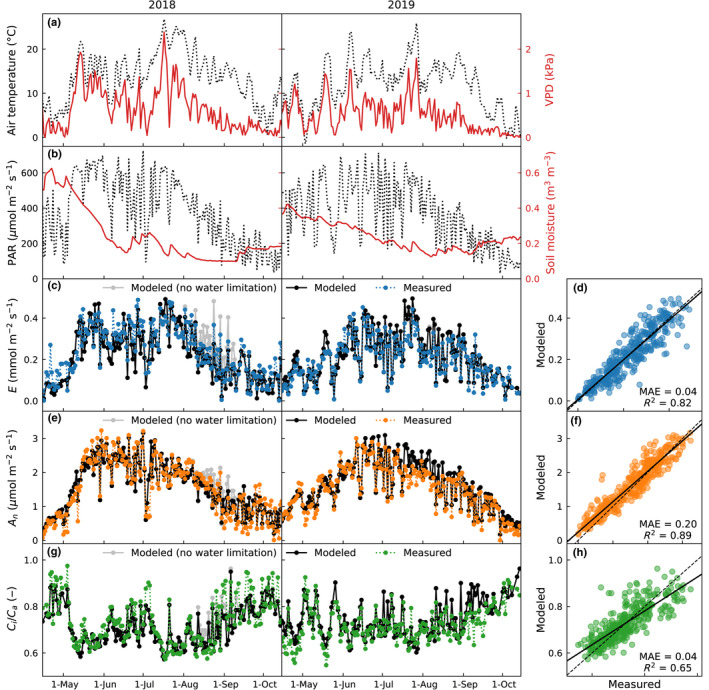
Environmental conditions and Scots pine shoot gas exchange during mid‐April to mid‐October 2018 and 2019. Daily mean (a) air temperature and vapor pressure deficit (VPD), and (b) photosynthetically active radiation (PAR) and soil moisture at 5 cm depth. Modeled and measured (c) transpiration, *E*, (e) net CO_2_ uptake, *A*_n_, and (g) intercellular to ambient CO_2_ concentration ratio, *C*_i_/*C*_a_. *E* and *A*_n_ are presented as daily means of times with observations and *C*_i_/*C*_a_ as daily medians for time when sun is above horizon. Model results neglecting the water limitation from soil moisture are shown in gray in (c, e, g). The fit between modeled and measured values is examined in (d, f, h), where the dashed line is 1 : 1 and the solid line the linear least squares regression. *R* ^2^ and MAE denote the coefficient of determination and mean absolute error, respectively.

The 2018 growing season was hotter and drier than 2019, as indicated by higher temperature and vapor pressure deficit (VPD), and lower top soil moisture (Fig. 1a,b). May–September precipitation was higher in 2018 (345 mm) than in 2019 (293 mm), but it was more evenly distributed in 2019 (not shown). During both years, temperature and VPD reached maximum values in July and PAR followed a bell‐shape reaching highest values in June (Fig. 1a,b). Shoot transpiration and net CO_2_ uptake had distinct seasonal cycles with highest daily fluxes reached in late June to mid‐July (Fig. 1c,e). Low soil moisture in August–September 2018 (Fig. 1b) limited photosynthesis, as shown by model runs with and without considering the water limitation (black vs gray lines in Fig. 1c,e). The value of *C*_i_/*C*_a_ was also affected by the water limitation (Fig. 1g). Overall, the model reproduced shoot gas exchange well (Fig. 1d,f,h). At half‐hourly timescale, *R* ^2^ for transpiration and net CO_2_ uptake were 0.86 and 0.90, respectively.

### The δ^18^O of needle water, sugars and water‐soluble carbohydrates

**Fig. 2:**
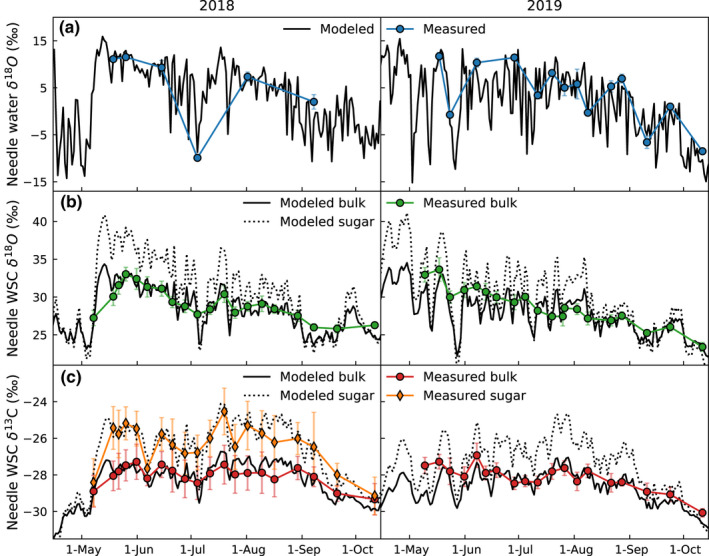
Modeled and measured (a) needle water δ^18^O, (b) δ^18^O of needle sugar and in bulk water‐soluble carbohydrates (WSC), and (c) δ^13^C of needle sugar and in bulk WSC of Scots pine. Modeled values are given as mean of 12:00–15:00 h, which corresponds to sampling interval. Error bars indicate the SDs of the five sampled trees.

**Fig. 3:**
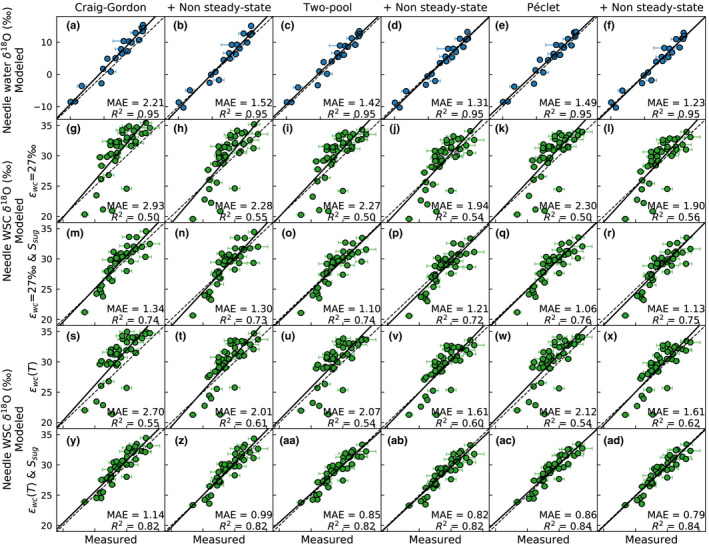
The fit between modeled and measured Scots pine (a–f) needle water δ^18^O, and (g–ad) δ^18^O of needle water‐soluble carbohydrates (WSC) with different needle water modeling approaches (see column titles) and model processes affecting δ^18^O of needle WSC. In (g–r) the biochemical fractionation factor, *ε*_wc_, is constant (27‰) and in (s–ad) *ε*_wc_ is temperature dependent following Sternberg & Ellsworth (2011). In (m–r) and (y–ad) the needle sugar pool size is set according to measurements, while in (g–l) and (s–x) its size is negligible. The dashed line is 1 : 1 and the solid line the linear least squares regression. *R* ^2^ and MAE denote the coefficient of determination and mean absolute error, respectively. Modeled values are given as mean of 12:00–15:00 h, which corresponds to sampling interval. Error bars indicate the SDs of the five sampled trees.

Measured needle water δ^18^O varied between −10‰ and 15‰ during the study period (Fig. 2a), whereas needle WSC δ^18^O was much less variable, 23–34‰ (Fig. 2b). Modeled needle water δ^18^O for the entire studied period (including periods between measurements) showed larger variability than measurements (Fig. 2a) because only a few sampling days occurred during low relative humidity (early July 2018 and late growing season of 2019). However comparing only data from sampling days, the model results (including Péclet effect with nonsteady state and a temperature‐dependent *ε*_wc_) well reproduced the seasonal variability in needle water and WSC δ^18^O (Fig. 2a,b), explaining 95% (Fig. 3f) and 84% (Fig. 3a,d) of their variability, respectively. In order to capture the measured needle WSC δ^18^O, the δ^18^O of pinitol was adjusted to 25‰, suggesting ^18^O‐depletion of pinitol in comparison to sugars. Hence, the variability of needle WSC δ^18^O was reduced compared to the variability modeled for needle sugar δ^18^O (Fig. 2b).

The different needle water model variants did not affect the amount of variation captured by the model (*R* ^2^ = 0.95, Fig. 3a–f). The level of needle water δ^18^O was best captured with either the two‐pool or the Péclet model under nonsteady state assumption (MAE = 1.23–1.31‰, Fig. 3d,f), whereas the Craig–Gordon model produced the highest overestimation (Fig. 3a,b). The nonsteady state assumption was also supported by the sampled diel needle water δ^18^O variation during May 23, 2019 (Fig. S7).

Accounting for the sugar pool size had a significant impact on the fit between modeled and measured δ^18^O of needle WSC. For example, with constant *ε*_wc_, *R* ^2^ improved from *c.* 0.5 (Fig. 3g–l) to 0.72–0.76 (Fig. 3m–r), when the sugar pool size was set according to measurements instead of a negligible size. The model fit improved further when the temperature‐dependent *ε*_wc_ was implemented, resulting in *R* ^2^ ranging from 0.82 to 0.84 (Fig. 3y–ad).

### The δ^13^C of needle sugars and water‐soluble carbohydrates

**Fig. 4:**
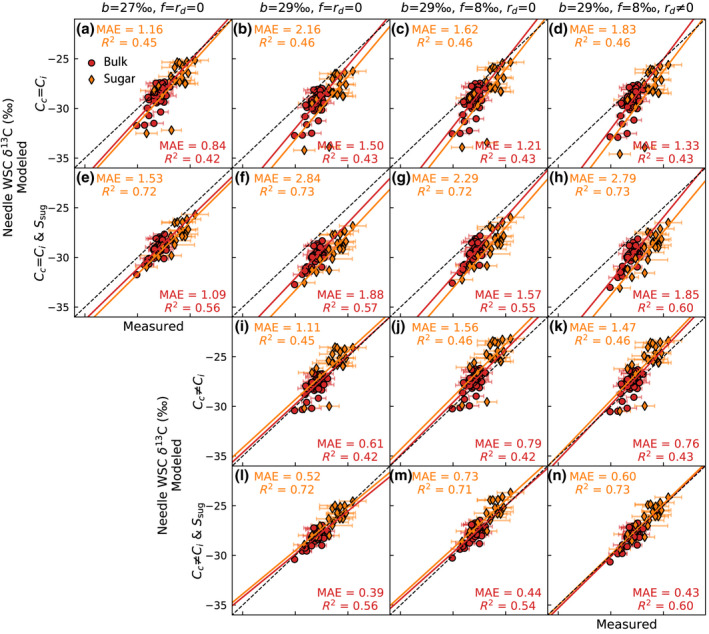
The fit between modeled and measured Scots pine δ^13^C of needle sugar and in bulk water‐soluble carbohydrates (WSC) with different model variants. The first column shows the commonly applied simple formulation, where the lower *b* implicitly accounts for all photosynthetic discrimination processes. The second column accounts only for fractionation by diffusion and carboxylation, the third adds photorespiration, and the last the effect of mitochondrial respiration. In (a–h) the models are based on *C*_c_ = *C*_i_ and in (i–n) the effect of mesophyll resistance is explicitly accounted for. Lastly, in (e–h) and (l–n) the needle sugar pool size is set according to measurements, while in (a–d) and (i–k) its size is negligible. The dashed line is 1 : 1 and the solid line the linear least squares regression. *R* ^2^ and MAE denote the coefficient of determination and mean absolute error, respectively. The upper corner values correspond to sugar and those in lower corner to bulk WSC. Modeled values are given as mean of 12:00–15:00 h, which corresponds to sampling interval. Error bars indicate the SDs of the five sampled trees.

The temporal variability of observed δ^13^C of needle bulk WSC was much smaller, with values ranging from −29‰ to −27‰ than that of needle sugar, with values ranging from −29‰ to −24.5‰ (Fig. 2c). This was expected due to the presence of pinitol with a near constant δ^13^C. The model explained 73% and 60% of the variability in δ^13^C of needle sugar and bulk WSC, respectively (Fig. 4n).

Evaluating the model variants for δ^13^C of needle sugar and WSC, we observe, as for δ^18^O, that accounting for the sugar pool size is important. The value of *R* ^2^ for needle sugar improved from 0.45–0.46 to 0.71–0.73, when the sugar pool size was accounted for (Fig. 4a–d, i–k vs e–h, l–n, respectively). Applying the model without explicitly accounting for mesophyll resistance captured 72–73% of the variability in observed needle sugar δ^13^C (Fig. 4e–h). However, in this case, the predicted values were generally too low (Fig. 4d–f) and only the simple model showed a slope close to unity (Fig. 4e). Explicitly accounting for mesophyll resistance (*C*_c_ ≠ *C*_i_) increased the predicted δ^13^C, which decreased MAE but did not improve *R* ^2^ (Fig. 4e–h vs l–n). Similarly, including photorespiration (*f* = 8‰) had no effect on the variability captured by the model (Fig. 4f,l vs g,m). Then again, including the effect of mitochondrial respiration (*r*_d_ ≠ 0) slightly improved *R* ^2^, especially for δ^13^C of WSC (Fig. 4g,m vs h,n), which covered two growing seasons, unlike δ^13^C of needle sugar (Fig. 2c). Overall, the best result (highest *R* ^2^ and lowest MAE) was obtained in Fig. 4(n) (*b* = 29‰, *f* = 8‰, *r*_d_ ≠ 0 and *C*_c_ ≠ *C*_i_), which corresponds to the time‐series shown in Fig. 2(c).

### Needle sugar pool size and formation period of isotopic signals

**Fig. 5:**
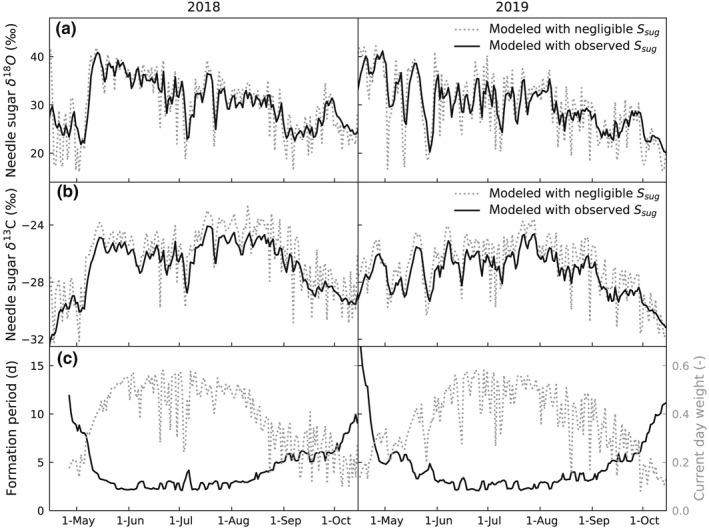
Modeled needle sugar (a) δ^18^O and (b) δ^13^C of Scots pine at 13:30 h with negligible sugar pool size and the observed sugar pool size. Panel (c) presents the formation period of the needle sugar pool isotopic signals and the weight of the current day assimilates in forming the isotopic signal of the needle sugar pool. The formation period and the current day weight were calculated assuming the sugar pool is well‐mixed and its size set equal to observed (see text).

As expected, modeling results with a negligible needle sugar pool size showed much larger variability than results obtained applying the observed sugar pool size (Fig. 5a,b). However, during times when day‐to‐day meteorological conditions were fairly stable, e.g. second half of May and early June 2018 (Fig. 1a,b), the role of the sugar pool size was less evident. Then again, the isotopic signal formed during occasional days of low VPD and PAR were not imprinting the sugar pool, because photosynthesis was typically low during these days and hence the role of current assimilates on shaping the isotopic composition of the sugar pool remained small (Fig. 5). The dynamics of the results obtained applying the observed sugar pool size were overall lagged and smoother compared to results with a negligible sugar pool size, especially in early and late growing season when the formation period of the sugar pool isotopic signal was longer (Fig. 5c). During mid‐growing season the sugar pool was formed of photosynthates assimilated over the past 48–52 h, while in early and late growing season the length of the formation period increased to over 5 d (Fig. 5c).

**Fig. 6:**
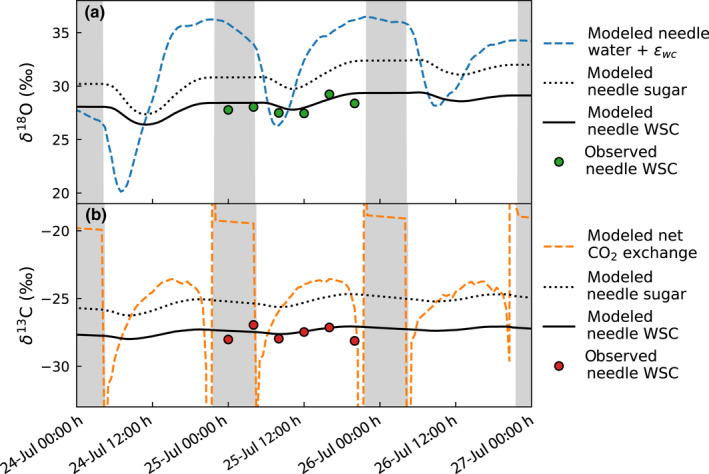
Modeled diurnal course of (a) δ^18^O and (b) δ^13^C signals in Scots pine needles during 24–26 July 2019 and measured needle isotopic signals of water‐soluble carbohydrates (WSC) sampled during 25 July 2019. In addition to modeled needle sugar and bulk WSC signals, (a) shows the isotopic signal of needle water + *ε*_wc_ (biochemical fractionation factor) and (b) the isotopic signal of net CO_2_ exchange. Gray‐shaded areas indicate night‐time. Extreme high and low values during the transition between day and night are caused by noise in model results when *kC*_a_ – *r*_d_ tends to zero (see Eqn 8).

The differences between the two modeling scenarios shown in Fig. 5(a,b) are not only caused by the combined effect of the sugar pool and day‐to‐day variation in meteorological conditions, but also the role the sugar pool has on the diurnal course of isotopic signals. Fig. 6 shows the modeled diurnal course of needle water δ^18^O + *ε*_wc_ and of δ^13^C of net CO_2_ exchange, which during daytime correspond to the sugar pool δ^18^O and δ^13^C, respectively, if the sugar pool size were negligible. In comparison to these, the modeled diurnal course of the sugar pool isotopic signals (applying the observed sugar pool size) are lagged and have a much lower amplitude. For δ^13^C this causes the 1–2‰ offset between the two scenarios in Fig. 5(b) as in the early afternoon δ^13^C of net CO_2_ exchange is higher compared to that of the sugar pool (Fig. 6b). For δ^18^O, the δ^18^O of needle water + *ε*_wc_ crosses the value of the sugar pool δ^18^O around midday (Fig. 6a) and hence Fig. 5(a) does not show a similar offset between the two modeling results as for δ^13^C. The δ^18^O and the δ^13^C of needle WSC measured from diurnal samples during July 25, 2019 support the low diurnal amplitude obtained by the model (Fig. 6a,b).

### Environmental and physiological signals

#### The δ^18^O and relative humidity

**Fig. 7:**
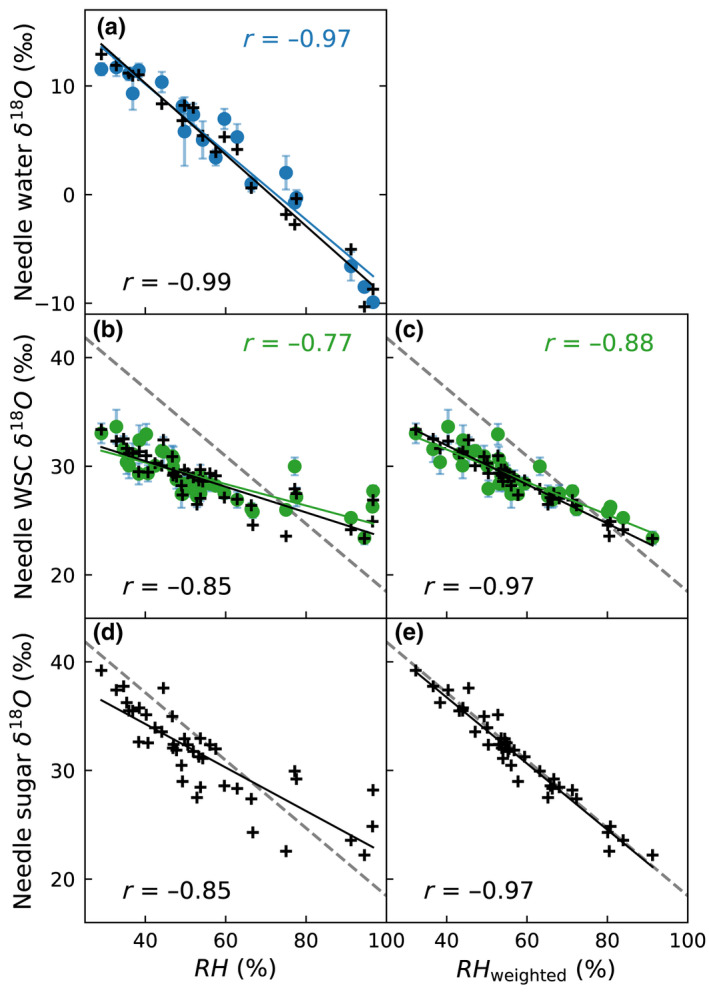
Relationship between relative humidity (RH) and (a) needle water δ^18^O, (b–c) δ^18^O of needle water‐soluble carbohydrates (WSC), and (d–e) needle sugar δ^18^O of Scots pine. In (a, b, d) RH corresponds to the sampling day (mean of midday ± 3 h) and in (c, e) RH is obtained by the weighting scheme outlined in the text. Colored dots and correlations (*r*) given in upper corner correspond to measurements, and plus‐signs and *r* given in lower corner to modeled values. The gray dashed line (b–e) indicates the relationship in (a) +27‰. Error bars indicate the SDs of the five sampled trees.

Both measured and predicted δ^18^O of needle water correlated strongly with the sampling day RH (Fig. 7a). The correlation between δ^18^O of needle WSC and RH was, however, weaker, and deviated from the expected relationship with RH observed for needle water δ^18^O offset by 27‰ (Fig. 7b). The modeled δ^18^O of needle sugar deviated less from this relationship but showed a lot of scatter (Fig. 7d). Both for modeled and measured δ^18^O, the correlations improved considerably, when we used the weighting scheme outlined earlier for RH to account for the sugar pool being an integration over time (Fig. 7b,d vs c,e). Also, accounting for the integration, the relationship between needle sugar δ^18^O and RH follows the expected line (Fig. 7e).

#### The δ^13^C and *C*_i_/*C*_a_

**Fig. 8:**
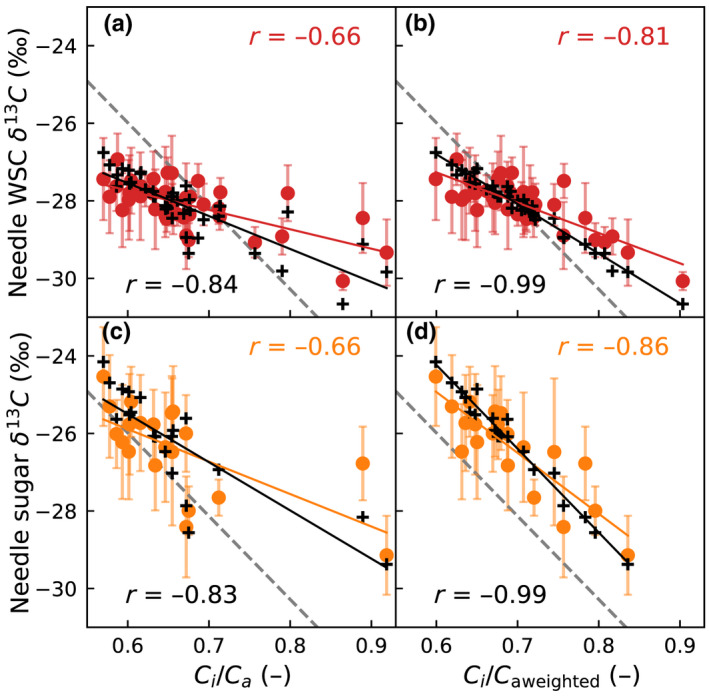
Relationship between modeled intercellular to ambient CO_2_ concentration ratio (*C*_i_/*C*_a_) and (a–b) δ^13^C of needle bulk WSC, and (c–d) needle sugar δ^13^C of Scots pine. In (a, c) *C*_i_/*C*_a_ corresponds to the sampling day (mean of midday + 3 h) and in (b, d) *C*_i_/*C*_a_ is obtained by the weighting scheme outlined in the text. Colored dots and correlations (*r*) given in upper corner correspond to measurements, and plus‐signs and *r* given in lower corner to modeled values. The gray dashed line corresponds to the simple model: ^13^Δ = *a*_s_ + (*b* – *a*_s_)*C*_i_/*C*_a_, with *b* = 27‰, *a*_s_ = 4.4‰ and δ^13^C of atmospheric CO_2_ set to its mean value −8.5‰. Error bars indicate the SDs of the five sampled trees.

Relationships between δ^13^C and *C*_i_/*C*_a_ showed a similar response as δ^18^O to RH; the variability of needle bulk WSC δ^13^C was reduced compared to that of needle sugar (Fig. 8a,b vs c,d), and the correlation strength increased once time integration was considered (Fig. 8a,c vs b,d).

The relationships in Fig. 8 are compared against the expected δ^13^C vs *C*_i_/*C*_a_ relationship (dashed gray line) defined following the simple model: ^13^Δ = *a*_s_ + (*b* – *a*_s_)*C*_i_/*C*_a_, with *b* = 27‰ and δ^13^C of atmospheric CO_2_ set to its mean value −8.5‰. The slope of this relationship is well represented by the observed and modeled needle sugar δ^13^C in Fig. 8(d), whereas Fig. 8(a–c) deviate from it because of neglecting the effect of time integration and/or because of the presence of pinitol. The miss‐match in level of *c.* 1.5‰ in Fig. 8(d) is in line with Fig. 4(e).

## Discussion

### Evidence for temperature‐dependent biochemical fractionation factor

Modeling of needle water δ^18^O indicated that its variation was strongly dictated by RH, as both RH (Fig. 7a) and the applied models (Fig. 3a–f) explained *c.* 95% of the variation of needle water δ^18^O (Roden & Ehleringer, 1999). Hereby at our study site, the variation in δ^18^O of water vapor and source water had only minor role, which supports the reconstruction of RH from tree ring δ^18^O records (Anderson *et al*., 1998; Wright & Leavitt, 2006). For water vapor δ^18^O we used IsoGSM data (Yoshimura et al., 2008), a good proxy at our study site (Fig. S3), thus not assuming vapor‐source water isotopic equilibrium (*R*_v_ = *R*_s_/*α* ^+^, Ogée *et al*., 2009). Leaf water δ^18^O is known to be rather insensitive to water vapor δ^18^O, because the ^18^O kinetic fractionation dominates over vapor‐source water isotopic disequilibrium (Cernusak *et al*., 2016). Then again, the minor role of source water δ^18^O was caused by its limited variability compared to the variability of evaporative ^18^O‐enrichment (Belmecheri *et al*., 2018). However, this does not necessarily hold, e.g. at sites where plant water sources vary between isotopically distinct precipitation and melting permafrost (Saurer *et al*., 2016).

In line with earlier studies, we found that the Craig–Gordon model overestimated needle water δ^18^O (Cernusak *et al*., 2016). We could not distinguish whether the two‐pool or the Péclet model provided a more suitable correction as, when calibrated, both models resulted in more or less equally good results (Fig. 3c–f). This has been typical also in earlier studies, but in some cases calibration has led to unrealistic parameter values (Song *et al*., 2013; Roden *et al*., 2015). The parameters obtained by calibration here, *f*_1_ = 0.93 and *L* = 30 mm, are reasonable in comparison to measured leaf anatomical characteristics (Roden *et al*., 2015; Timofeeva *et al*., 2020). However, there exist uncertainties related to the calibrated parameter values due to, e.g. the assumption of leaf temperature being equal to air temperature (Ogée *et al*., 2009; Cernusak *et al*., 2016) and the source of diffusional fractionation factors (Merlivat, 1978; Cappa *et al*., 2003).

Based on the diurnal course of needle water δ^18^O (Fig. S7), we recognized the need for the nonsteady‐state model (Barnard *et al*., 2007; Gessler *et al*., 2013). However, its impact on δ^18^O of needle sugar was less critical (Fig. 3ac vs ad), because during times of highest assimilation δ^18^O of needle water was close to the steady‐state solution. This suggests steady‐state leaf water models could be sufficient in predicting plant cellulose δ^18^O (Ogée *et al*., 2009; Hirl *et al*., 2021), simplifying model structure and parametrization needs.

One of our key findings was that implementing the temperature‐dependent biological fractionation factor (*ε*_wc_), following results from laboratory experiments by Sternberg & Ellsworth (2011), improved predictions of needle WSC δ^18^O notably (Fig. 3m–r vs y–ad). So far only one study (Hirl *et al*., 2021) has shown such evidence in field conditions and our study is the first to show this for trees. For our study site, with May–September temperatures varying between −2.9 and 30.7°C, *ε*_wc_ ranged from 34.6 to 25.4‰. Although this has important implications to predicting plant δ^18^O signals, here the implemented temperature dependence did not clearly interfere with the dominant effect of RH in driving needle sugar δ^18^O variability (Fig. 7e). The influence of the temperature‐dependent *ε*_wc_ is however expected to be critical in climate and leaf temperature reconstruction studies spanning across various temperature zones. For example, as shown by Sternberg & Ellsworth (2011), the temperature‐dependent *ε*_wc_ may explain the unexpected strong correlation between mean annual temperature and cellulose ^18^O‐enrichment found across 39 tree species at 25 sites by Helliker & Richter (2008).

A challenge of this study was that δ^18^O was not measured for needle sugars, but only for bulk WSC. We assumed that WSC consisted of sugars reflecting needle water δ^18^O and pinitol with a constant δ^18^O. To capture the observed WSC δ^18^O, pinitol δ^18^O was adjusted to 25‰, which is reasonable compared to the 22‰ reported for pinitol in Siberian larch (Lehmann *et al*., 2017). However, Lehmann *et al*. (2017) also found differences between δ^18^O of sucrose (most ^18^O‐enriched), fructose and glucose, thus there remains some uncertainty in whether it is only the relatively high amounts of ^18^O‐depleted pinitol that causes the reduced variation in δ^18^O of needle WSC. While the modeling suggested ^18^O‐depleted pinitol as one plausible explanation, further compound specific δ^18^O analyses are needed to verify our finding.

### Mesophyll resistance has important role in determining the level of needle sugar δ^13^C

The *C*_i_/*C*_a_ was the dominant driver of δ^13^C variation in the modeled needle carbohydrate pools as expected (Farquhar *et al*., 1982). Increasing model complexity provided only limited improvement to the explained variation. Interestingly, we found that the simple model with the bulk fractionation factor *b* = 27‰ (Fig. 4e; Farquhar *et al*., 1982; Ubierna & Farquhar, 2014) captured the variability almost as well as the comprehensive model (Fig. 4n) and only showed a fairly constant offset of 1.5‰. This indicates that changes in *C*_i_/*C*_a_ can be derived from changes in needle sugar δ^13^C using the simple model (^13^Δ = 4.4 + (27 – 4.4)*C*_i_/*C*_a_). However, explicitly accounting for mesophyll resistance (i.e. *C*_c_ ≠ *C*_i_), was required to capture the absolute level of observed δ^13^C (Warren *et al*., 2003; Ubierna & Marshall, 2011; Gentsch *et al*., 2014). The chosen description for mesophyll conductance (Eqn S9 in Methods S1) also played a key role as it defines *C*_c_ ≈ 0.8*C*_i_ (Fig. S8a). By contrast, applying a constant mesophyll conductance (e.g. Wingate *et al*., 2007; Ogée *et al*., 2009) or a constant ratio between stomatal and mesophyll conductance (e.g. Vernay *et al*., 2020) result in *C*_c_ approaching *C*_i_ at high *C*_i_ (Fig. S8b,c), which would have led to poorer model agreement, as there was a miss‐match in δ^13^C values along the whole range of *C*_i_/*C*_a_ (Fig. 8d). This underlines the need for better understanding on the variability of mesophyll conductance at intra‐seasonal scale.

### Need to account for needle sugar pool size to predict its isotopic composition

For both δ^18^O and δ^13^C, results indicated clearly that the needle sugar pool size has a crucial role in reducing its day‐to‐day (Fig. 5a,b) and sub‐daily (Fig. 6) variation of isotopic composition compared to that of new assimilates. In line with the ^13^C‐pulse‐labeling study on *Pinus pinaster* by Desalme *et al*. (2017), we suggest that the signal of the sugar pool was composed of sugars assimilated over the past 48 h to more than 5 d depending mostly on the time of the growing season (Fig. 5c). While time lags and attenuated diurnal patterns between, e.g. leaf water and leaf organic matter δ^18^O are commonly recognized (Barnard *et al*., 2007; Gessler *et al*., 2013), their causes have not been quantitatively attributed to the sugar pool size, as done here using dynamic modeling. Examining our results at different timescales provides vital information for the interpretation of leaf‐level isotopic data. For example: (1) the difference between instant leaf water δ^18^O and leaf sugar δ^18^O seldom equals *ε*_wc_ (Figs 5a, 6a; Gessler *et al*., 2013; Lehmann *et al*., 2017); (2) online measured δ^13^C of net CO_2_ exchange (e.g. Wingate *et al*., 2007; Schiestl‐Aalto *et al*., 2021) is expected to be higher than δ^13^C of needle sugar sampled in early afternoon (Fig. 6b); and (3) sudden day‐to‐day variations in meteorological conditions are not strongly reflected in isotopic signals of the needle sugar pool (Fig. 5a,b). Explicitly attributing such phenomena to the simple accumulation and mixing of new assimilates in the needle sugar pool is highly relevant to avoid miss‐interpreting fractionation processes in leaves.

The applied assumption that needle sugars form one well‐mixed pool of constant size may be debatable. It has been suggested that sucrose appears both in a fast transport pool and a slow transport pool (Brauner *et al*., 2014; Bögelein *et al*., 2019), which would reduce the variation in needle sugar δ^13^C further compared to our predictions. The concept of various transport pools might become critical when predicting isotopic compositions further downstream from leaves (e.g. phloem or tree ring cellulose), but here at leaf‐level its role could not be identified. Also, the concentration of needle sugars is expected to vary diurnally (Liesche *et al*., 2021) and seasonally with increased levels at the start and end of growing season (Schiestl‐Aalto *et al*., 2019). With high needle sugar concentrations, typical for trees growing under cold winters (Fig. S5; Kagawa *et al*., 2006; Rinne *et al*., 2015), we can however expect the diurnal variation in concentration to be small compared to the absolute concentration value. For the seasonal variation, our data showed no clear pattern (Fig. S5), plausibly indicating that during the active growing season, which is the crucial period for tree ring formation, the assumption of constant sugar pool size is sufficient.

### Time integration and sample composition critical for retrieving environmental/physiological signals from needle δ^18^O and δ^13^C

The correlations between δ^18^O and RH, and δ^13^C and *C*_i_/*C*_a_ further underlined the need to account for the needle sugar pool being an integration over time (Figs 7b–e, 8). The correlations were clearly weaker, when examined against sampling day RH and *C*_i_/*C*_a_ compared to RH and *C*_i_/*C*_a_ weighted according to past assimilation and mixing in the sugar pool. The correlation strength between δ^13^C and *C*_i_/*C*_a_ did not clearly differ for needle WSC and needle sugars (Fig. 8), which supports our assumption on constant pinitol share and pinitol δ^13^C. The main difference between needle WSC and needle sugars was the slope of the relationships, which for WSC deviated from the expected relationships due to the presence of depleted pinitol (Figs 7c, 8b). This emphasizes the need to know the sample composition, in order to draw conclusions about the magnitude of underlying environmental/physiological changes causing the variation of the isotopic signals (Stokes *et al*., 2010; Tarin *et al*., 2020). If the constant share and isotopic signal of pinitol are known, one can estimate changes in RH or *C*_i_/*C*_a_ from bulk WSC δ^18^O or δ^13^C, respectively, as the isotopic signal of needle sugar is a linear function of that of bulk WSC (Eqn 7).

Examining the environmental/physiological signals both for model results and observations provided means to explain the phenomena behind the present relationships or the lack of them (see also Hirl *et al*., 2021). Such evaluation is valuable and has potential to bridge the gap between empirical studies, focused on the climatic signals of tree ring isotopic records, and mechanistic modeling studies, in order to further advance the interpretation of isotopic signals in trees.

## Competing interests

None declared.

## Author contributions

KL, SL and KTR‐G designed the study. KTR‐G planned the sampling scheme and led the analysis of isotopic data. YT, PS‐A, ES and KTR‐G conducted the fieldwork, YT conducted laboratory preparation of samples, and ES, MS and AK performed the isotope analysis. PK processed the shoot chamber data. KL analyzed the data with assistance from YT and PK, and built the model and run it with guidance from JO, SL and PS‐A. All authors participated in interpreting the results and writing the manuscript in the lead of KL.

## Supporting information


**Fig. S1** Measured needle sucrose δ^13^C against measured needle sugar (sucrose + glucose + fructose) δ^13^C in current‐year needles and 1‐yr‐old needles.
**Fig. S2** Measured isotopic composition of needle water‐soluble carbohydrates in current‐year needles and 1‐yr‐old needles, and a combined data series over the two needle generations.
**Fig. S3** Measured δ^18^O of atmospheric water vapor against corresponding values predicted by IsoGSM.
**Fig. S4** Modeled and measured source (twig) water δ^18^O and observed soil water δ^18^O.
**Fig. S5** Measured concentrations of needle water‐soluble carbohydrates and measured ratio of needle pinitol to needle sugar (sucrose + glucose + fructose) concentrations in current‐year needles and 1‐yr‐old needles.
**Fig. S6** Measured water content of 1‐yr‐old needles.
**Fig. S7** Modeled and measured diurnal course of needle water δ^18^O.
**Fig. S8** Modeled relationship between CO_2_ mole fraction in chloroplast and intercellular spaces using different descriptions for mesophyll conductance.
**Methods S1** Modeling shoot gas exchange.
**Methods S2** Derivation of model for ^13^C‐discrimination of net CO_2_ exchange (Eqn 8).
**Methods S3** Modeling source water δ^18^O.
**Methods S4** Derivation of Eqns (Eqn 10), (Eqn 11), (Eqn 12).
**Table S1** Parameter values applied for shoot gas exchange modeling.
Please note: Wiley Blackwell are not responsible for the content or functionality of any Supporting Information supplied by the authors. Any queries (other than missing material) should be directed to the *New Phytologist* Central Office.

## Data Availability

The model code was written in Python and is available at https://github.com/LukeEcomod/LeafIsotopes with an example run corresponding to this study. Environmental and eddy‐covariance data for the study site (SMEAR II Hyytiälä forest) can be obtained from https://smear.avaa.csc.fi/. The isotopic and concentration data are available on request to the corresponding author.
