# Supplementary material for: Explicitly accounting for needle sugar pool size crucial for predicting intra‐seasonal dynamics of needle carbohydrates δ18O and δ13C
Source: New Phytol. 2022 Jun 24;236(6):2044–60. doi: 10.1111/nph.18227 (PMC9795997; doi:10.1111/nph.18227)
Supplement: Supplementary file 1 — Fig. S1 Measured needle sucrose δ13C against measured needle sugar (sucrose + glucose + fructose) δ13C in current‐year needles and 1‐yr‐old needles. Fig. S2 Measured isotopic composition of needle water‐soluble carbohydrates in current‐year needles and 1‐yr‐old needles, and a combined data series over the two needle generations. Fig. S3 Measured δ18O of atmospheric water vapor against corresponding values predicted by IsoGSM. Fig. S4 Modeled and measured source (twig) water δ18O and observed soil water δ18O. Fig. S5 Measured concentrations of needle water‐soluble carbohydrates and measured ratio of needle pinitol to needle sugar (sucrose + glucose + fructose) concentrations in current‐year needles and 1‐yr‐old needles. Fig. S6 Measured water content of 1‐yr‐old needles. Fig. S7 Modeled and measured diurnal course of needle water δ18O. Fig. S8 Modeled relationship between CO2 mole fraction in chloroplast and intercellular spaces using different descriptions for mesophyll conductance. Methods S1 Modeling shoot gas exchange. Methods S2 Derivation of model for 13C‐discrimination of net CO2 exchange (Eqn 8). Methods S3 Modeling source water δ18O. Methods S4 Derivation of Eqns (Eqn 10), (Eqn 11), (Eqn 12). Table S1 Parameter values applied for shoot gas exchange modeling. Please note: Wiley Blackwell are not responsible for the content or functionality of any Supporting Information supplied by the authors. Any queries (other than missing material) should be directed to the New Phytologist Central Office. [file NPH-236-2044-s001.pdf]

New Phytologist

New Phytologist Supporting Information

Article title: **Explicitly accounting for needle sugar pool size crucial for predicting intra-seasonal dynamics of needle carbohydrates $\delta^{18}O$ and $\delta^{13}C$**

Authors: Kersti Leppä, Yu Tang, Jérôme Ogée, Samuli Launiainen, Ansgar Kahmen, Pasi Kolari, Elina Sahlstedt, Matthias Saurer, Pauliina Schiestl-Aalto, Katja T. Rinne-Garmston

Article acceptance date: 7 May 2022

The following Supporting Information is available for this article:

- **Fig. S1** Measured needle sucrose $\delta^{13}C$ against measured needle sugar (sucrose+glucose+fructose) $\delta^{13}C$ in current-year needles and one-year-old needles.
- **Fig. S2** Measured isotopic composition of needle water-soluble carbohydrates in current-year needles and one-year-old needles, and a combined data series over the two needle generations.
- **Fig. S3** Measured $\delta^{18}O$ of atmospheric water vapor against corresponding values predicted by IsoGSM.
- **Fig. S4** Modeled and measured source (twig) water $\delta^{18}O$ and observed soil water $\delta^{18}O$.
- **Fig. S5** Measured concentrations of needle water-soluble carbohydrates and measured ratio of needle pinitol to needle sugar (sucrose+glucose+fructose) concentrations in current-year needles and one-year-old needles.
- **Fig. S6** Measured water content of one-year-old needles.
- **Fig. S7** Modeled and measured diurnal course of needle water $\delta^{18}O$.
- **Fig. S8** Modelled relationship between CO_2 mole fraction in chloroplast and intercellular spaces using different descriptions for mesophyll conductance.
- **Table S1** Parameter values applied for shoot gas exchange modeling
- **Methods S1** Modeling shoot gas exchange
- **Methods S2** Derivation of model for ^{13}C-discrimination of net CO_2 exchange (Eq. 8)
- **Methods S3** Modeling source water $\delta^{18}O$
- **Methods S4** Derivation of Eqs. 10–12

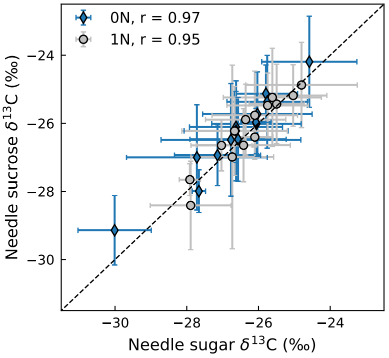

Fig. S1 Measured needle sucrose δ^{13}C against measured needle sugar (sucrose+glucose+fructose) δ^{13}C in current-year needles (0N) and one-year-old needles (1N) of Scots pine. r denotes Pearson's correlation and error bars indicate the standard deviation of the five sampled trees.

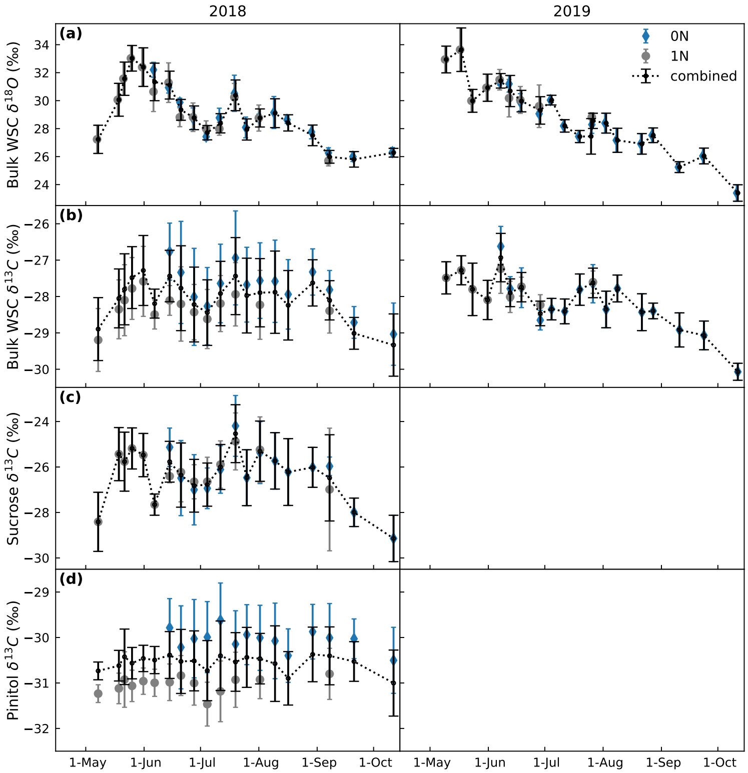

Fig. S2 Measured isotopic composition of needle water-soluble carbohydrates (WSC) in current-year needles (0N) and one-year-old needles (1N) of Scots pine, and a combined data series over the two needle generations. (**a**) δ^{18}O of bulk WSC, (**b**) δ^{13}C of bulk WSC, (**c**) δ^{13}C of sucrose, and (**d**) δ^{13}C of pinitol. Error bars indicate the standard deviations of the five sampled trees.

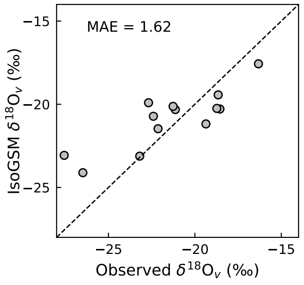

Fig. S3 δ^{18}O of atmospheric water vapor measured at the experimental site against corresponding values predicted by IsoGSM (Yoshimura et al., 2008). The dashed line is 1:1 and MAE denotes the mean absolute error between the datasets.

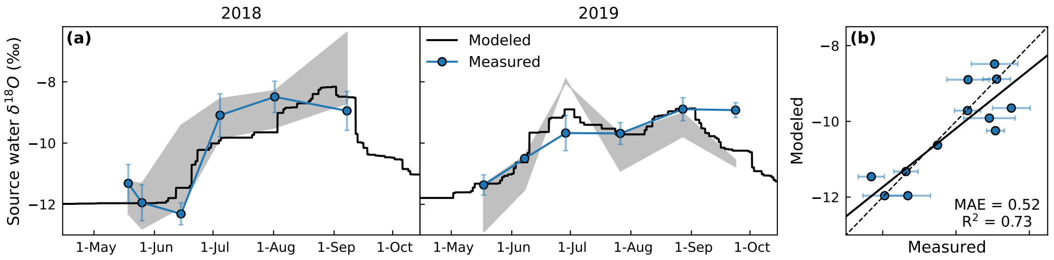

Fig. S4 (**a**) Time course of modeled and measured source (twig) water δ^{18}O of Scots pine and (**b**) modeled against measured values. The dashed line is 1:1 and the solid line the linear least squares regression. R^2 and MAE denote the coefficient of determination and mean absolute error, respectively. Error bars present standard deviation of measurements from five trees. The range of observed soil water δ^{18}O between 0.02 m and 0.1 m is shown as grey shaded area.

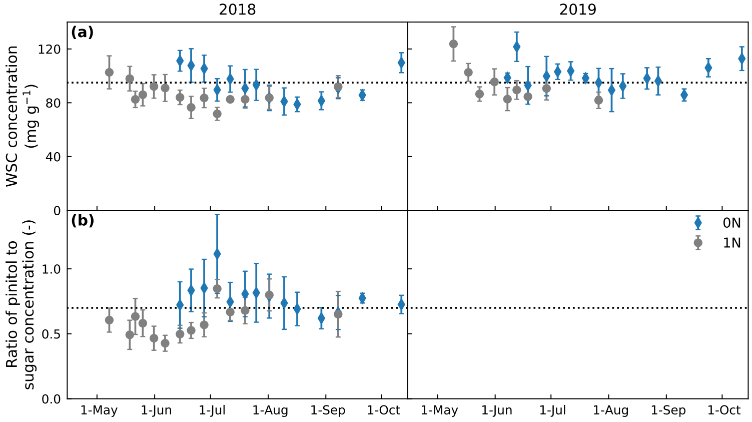

Fig. S5 (**a**) Measured concentrations of needle water-soluble carbohydrates (WSC) and (**b**) measured ratio of needle pinitol to needle sugar (sucrose+glucose+fructose) concentrations in current-year needles (0N) and one-year-old needles (1N) of Scots pine. The dotted lines indicate the values used in the modeling. Error bars indicate the standard deviations of the five sampled trees.

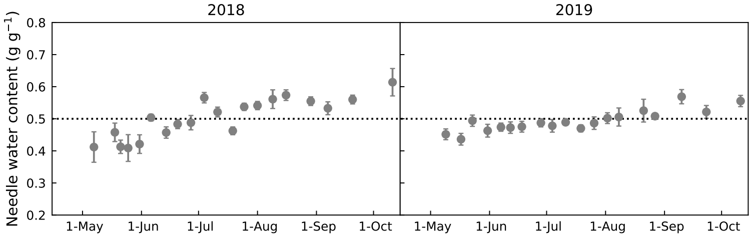

Fig. S6 Water content of one-year-old needles of Scots pine obtained by measuring the fresh weight of needle samples and the dry weight of needle samples after oven-drying. The dotted lines indicate the value used in the modeling. Error bars indicate the standard deviations of the five sampled trees.

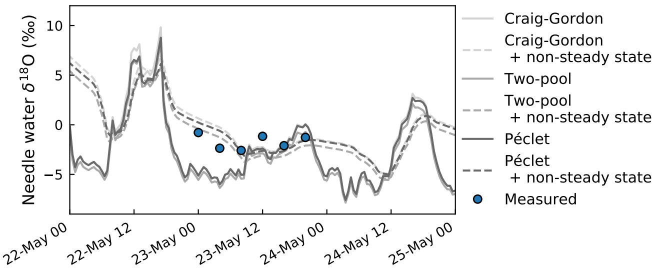

Fig. S7 Modeled diurnal course of needle water $\delta^{18}O$ during 22 to 24 May 2019 and measured values for Scots pine sampled during 23 May 2019.

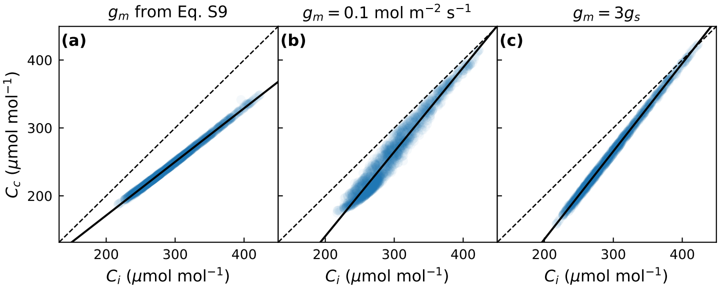

Fig. S8 Modelled relationship between CO_2 mole fraction in chloroplast (C_c) and intercellular spaces (C_i) when using (**a**) mesophyll conductance defined by Eq. S9, (**b**) constant mesophyll conductance and (**c**) a constant ratio between stomatal and mesophyll conductance. The dashed line is 1:1 and the solid line the linear least squares regression.

Table S1 Parameter values applied for shoot gas exchange modeling

Parameter	Unit[a]	Description	Value	Source
V_{cmax25}[b]	µmol m^{-2} s^{-1}	maximum carboxylation capacity at 25°C	32	calibrated
J_{max25}[b]	µmol m^{-2} s^{-1}	maximum electron transport rate at 25°C	$2 \times V_{cmax25}$	Kattge and Knorr (2007)
r_{d25}[c]	µmol m^{-2} s^{-1}	mitochondrial respiration rate at 25°C	$0.014 \times V_{cmax25}$	night-time shoot chamber data
θ	-	curvature of the response of electron transport to irradiance, Eq. S6	0.7	Launiainen et al. (2015)
I	µmol m^{-2} s^{-1}	photosynthetically active radiation (PAR) effectively absorbed by photosystem II, Eq. S6	$0.05 \times$ PAR	calibrated (note that PAR given by ground area)
Γ_*	µmol m^{-2} s^{-1}	CO_2 compensation point in absence of r_d	-	Bernacchi et al. (2001)
K_c, K_o	µmol m^{-2} s^{-1}	Michaelis–Menten constants for CO_2 and O_2	-	Bernacchi et al. (2001)
T_0	°C	threshold for delayed temperature, Eq. S7	-3.1	Mäkelä et al. (2008)
S_{max}	°C	level at which delayed temperature reaches saturation, Eq. S7	14.2	Mäkelä et al. (2008)
τ	days	time constant for delayed temperature	18	calibrated
g_b	mol m^{-2} s^{-1}	boundary layer conductance for CO_2	1.5	Wingate et al. (2007)
$g_{s,0}$	mol m^{-2} s^{-1}	stomatal model parameter, Eq. S8	0.001	shoot chamber data
$g_{s,1}$	kPa$^{0.5}$	stomatal mode parameter, Eq. S8	2.0	shoot chamber data
$g_{s,night}$	mol m^{-2} s^{-1}	night-time stomatal conductance	0.003	night-time shoot chamber data
$g_{m,1}$	-	mesophyll conductance parameter, Eq. S9	4.5	calibrated
$g_{m,night}$	mol m^{-2} s^{-1}	minimum value for mesophyll conductance	0.01	
b_0	-	water stress parameter for $g_{s,1}$ and V_{cmax25}, Eq. S10	0.31, 0.39	Launiainen et al. (2022)
b_1	-	water stress parameter for $g_{s,1}$ and V_{cmax25}, Eq. S10	3.0, 0.83	Launiainen et al. (2022)

[a]all area-based units refer to all-sided leaf area; [b]parameters of temperature response functions (Medlyn et al., 2002) adopted from Tarvainen et al. (2013); [c]temperature response function (Bernacchi et al., 2001) fitted to night-time shoot chamber data

Methods S1 Modeling shoot gas exchange

The exchange of water vapor (E, mol m^{-2} s^{-1}) and CO_2 (A_n, µmol m^{-2} s^{-1}) between the shoot and air follow

$$E = 1.6 g_b (w_s - w_a) = 1.6 g_s (w_i - w_s) \tag{S1}$$

$$A_n = g_b (C_a - C_s) = g_s (C_s - C_i) = g_m (C_i - C_c) \tag{S2}$$

where g_b, g_s and g_m (mol m^{-2} s^{-1}) are boundary layer, stomatal, and mesophyll conductance for CO_2, respectively; 1.6 is the ratio of molecular diffusivity of water vapor to that of CO_2 in air; w_a, w_s and w_i (mol mol^{-1}) are mole fractions of water vapor in the atmosphere, at the leaf surface and inside the leaf, respectively; C_a, C_s, C_i and C_c (µmol mol^{-1}) are CO_2 mole fractions in the atmosphere, at the leaf surface, inside the leaf, and in the chloroplast, respectively. In the model, we assume vapor pressure inside the leaf is saturated and that the leaf is at air temperature.

Following the photosynthesis model of Farquhar et al. (1980), net leaf CO_2 exchange is given as

$$A_n = \min(A_c, A_j) - r_d \tag{S3}$$

where A_c and A_j (µmol m^{-2} s^{-1}) are Rubisco- and RuBP regeneration-limited assimilation rates, respectively, and r_d (µmol m^{-2} s^{-1}) is mitochondrial respiration. The Rubisco-limited rate is defined as

$$A_c = (1 - \Gamma_*/C_c) \frac{V_{cmax} C_c}{C_c + K_c (1 + O/K_o)} \tag{S4}$$

where V_{cmax} (µmol m^{-2} s^{-1}) is the maximum rate of Rubisco activity, Γ_* (µmol mol^{-1}) is the CO_2 compensation point in the absence of mitochondrial respiration, K_c and K_o (µmol mol^{-1}) are the Michaelis–Menten constants for CO_2 and O_2, respectively, and O is the oxygen mixing ratio (2.1×10^5 µmol mol^{-1}). The RuBP regeneration-limited rate follows

$$A_j = (1 - \Gamma_*/C_c) \frac{J C_c}{4(C_c + 2\Gamma_*)} \tag{S5}$$

where the irradiance response of the rate of electron transport J (µmol m^{-2} s^{-1}) is modeled by

$$\theta J^2 - (I + J_{max}) J + I J_{max} = 0 \tag{S6}$$

where θ (-) is the curvature of the response of electron transport to irradiance, J_{max} (µmol m^{-2} s^{-1}) is the potential electron transport rate, and I (µmol m^{-2} s^{-1}) is the photosynthetically active radiation (PAR) effectively absorbed by photosystem II. In Eqs. S4–S5, the term $(1 - \Gamma_*/C_c)$ is used to account for CO_2 release through photorespiration, i.e. carboxylation $V_c = \min(A_c, A_j)/(1 - \Gamma_*/C_c)$ and photorespiration $F = V_c \Gamma_*/C_c$.

The temperature dependency of V_{cmax} and J_{max} have the general form given in Medlyn et al. (2002) and r_d that in Bernacchi et al. (2001). Additionally, maximum carboxylation capacity V_{cmax25}, maximum electron transport rate J_{max25}, and mitochondrial respiration rate r_{d25} at a reference temperature of 25°C are assumed to vary with seasonal cycle of photosynthetic capacity following a delayed temperature model (Mäkelä et al., 2008)

$$f_{pheno} = \min\left(\max\left(\frac{S - T_0}{S_{max} - T_0}, 1\right), 0\right) \tag{S7}$$

where S_{max} and T_0 (°C) are parameters, and the delayed temperature S (°C) is solved from $dS/dt = (T_{daily} - S)/\tau$,

where T_{daily} (°C) is mean daily ambient temperature and τ (days) is the time constant of the delay process.

As defined by Eq. S2, A_n is additionally limited by stomatal and mesophyll conductance. Stomatal conductance (g_s, mol m^{-2} s^{-1}) is defined by the unified stomatal model relying on the principal that stomata act to minimize the amount of water used per unit carbon gained (Medlyn et al., 2011)

$$g_s = g_{s,0} + \left(1 + \frac{g_{s,1}}{\sqrt{D}}\right)\frac{A_n}{C_s} \tag{S8}$$

where $g_{s,0}$ (mol m^{-2} s^{-1}) and $g_{s,1}$ (kPa$^{0.5}$) are parameters and D (kPa) is vapor pressure deficit. Eq. S8 describes stomatal behavior when photosynthesis is active, therefore an additional parameter $g_{s,night}$ (mol m^{-2} s^{-1}) is defined as a lower limit for g_s. $g_{s,night}$ has an important role in the non-steady-state solution of leaf water δ^{18}O (Ogée et al., 2009).

The behavior of mesophyll conductance (g_m, mol m^{-2} s^{-1}) is less well understood, but its role in ^{13}C-discrimination is widely recognized (Medlyn et al., 2017). Here, we used a g_m description suggested by Dewar et al. (2018) and applied successfully for Scots pine by Schiestl-Aalto et al. (2021)

$$g_m = g_{m,1}\frac{A_n}{C_c - \Gamma_*} \tag{S9}$$

where $g_{m,1}$ (-) is a fitted parameter. Again, Eq. S9 describes only conditions when photosynthesis is active, thus $g_{m,night}$ (mol m^{-2} s^{-1}) is defined as a lower limit for g_m.

To account for water stress (Zhou et al., 2013; Kellomaki and Wang, 1996), V_{cmax25} and $g_{s,1}$ (Eq. S8) were adjusted as non-linear functions of relative plant extractable water (Launiainen et al., 2022)

$$f_w = \min\left(1.0, \left(\frac{Rew}{b_0}\right)^{b_1}\right) \tag{S10}$$

where $Rew = (\theta - \theta_r)/(\theta_s - \theta_r)$ and b_0 and b_1 are fitting parameters. The soil water content θ (m^3 m^{-3}) was measured at ca. 5cm depth in the mineral soil, where field capacity and residual water content were θ_s = 0.30 m^{3}m^{-3} and θ_r = 0.03 m^{3}m^{-3}, respectively. b_0 and b_1 were adopted from Launiainen et al. (2022), who fitted the parameters to shoot chamber data from the same site during drought year 2006 .

All parameters values applied in the modeling of shoot gas exchange at the study site are listed in Table S1.

Methods S2 Derivation of model for ^{13}C-discrimination of net CO_2 exchange (Eq. 8)
The starting point is Eq. A2.6 in Appendix II of Wingate et al. (2007):

$$^{13}\Delta = \frac{kC_a}{k(C_a - \Gamma_*) - r_d}\left(a_s + (b - a_s)\frac{C_i}{C_a} - (^{13}\Delta + f)\frac{\Gamma_*}{C_a} - \frac{r_d}{kC_a}\left(1 - \frac{R_{sug}}{R_a}(1 - e)\right)\right) \tag{S11}$$

where $R_{substrate}$ of Wingate et al. (2007) is replaced by R_{sug} as that is our substrate for mitochondrial respiration. Solving Eq. S11 for $^{13}\Delta$, which now appears on both sides of equation, results in:

$$^{13}\Delta = \frac{kC_a}{kC_a - r_d}\left(a_s + (b - a_s)\frac{C_i}{C_a} - f\frac{\Gamma_*}{C_a}\right) - \frac{r_d}{kC_a - r_d}\left(1 - \frac{R_{sug}}{R_a}(1 - e)\right) \tag{S12}$$

Here, the first term accounts for diffusion through stomata, carboxylation and photorespiration. However, as in Wingate et al. (2007) this part can be easily extended to cover also diffusion through the leaf boundary layer and

mesophyll:

$$^{13}\Delta = \frac{kC_a}{kC_a - r_d}\left(a_b\frac{C_a - C_s}{C_a} + a_s\frac{C_s - C_i}{C_a} + a_m\frac{C_i - C_c}{C_a} + b\frac{C_c}{C_a} - f\frac{\Gamma_*}{C_a}\right) - \frac{r_d}{kC_a - r_d}\left(1 - \frac{R_{sug}}{R_a}(1-e)\right) \quad (S13)$$

The derivation in Wingate et al. (2007) was based on the definition $A_n'/A_n = R_a(1 - {}^{13}\Delta)$ and $r_d'/r_d = R_{sug}(1 - e)$ for discrimination (where A_n'/A_n and r_d'/r_d are the isotopic ratios of net CO_2 exchange and dark respiration, respectively). Because of this, Eq. S13 reduces to $R_a(1 - {}^{13}\Delta) = R_{sug}(1 - e)$ in dark, when $k = 0$, representing discrimination by dark respiration (r_d'/r_d). However, discrimination is more commonly defined as $A_n'/A_n = R_a/(1 + {}^{13}\Delta)$ and $r_d'/r_d = R_{sug}/(1 + e)$ (Farquhar et al., 1989), and this is the definition that is used for ^{18}O-discrimination. Deriving the classical discrimination equation (Farquhar et al., 1982) based on this formulation is less complex and requires fewer second-order approximations (Farquhar et al., 1989). To be in line with the more common formulation for discrimination, and use consistent definitions of discrimination for both ^{13}C and ^{18}O, the r_d-term (last term in Eq. S13) needs to be modified so that in dark Eq. S13 reduces to $R_a/(1 + {}^{13}\Delta) = R_{sug}/(1 + e)$. As a function of $^{13}\Delta$, this is expressed as:

$$^{13}\Delta = \frac{R_a}{R_{sug}}(1+e) - 1 \quad (S14)$$

where the right-hand side corresponds to the modified r_d-term of Eq. 8:

$$^{13}\Delta = \frac{kC_a}{kC_a - r_d}\left(a_b\frac{C_a - C_s}{C_a} + a_s\frac{C_s - C_i}{C_a} + a_m\frac{C_i - C_c}{C_a} + b\frac{C_c}{C_a} - f\frac{\Gamma_*}{C_a}\right) - \frac{r_d}{kC_a - r_d}\left(\frac{R_a}{R_{sug}}(1+e) - 1\right) \quad (S15)$$

Although inserting Eq. S14 into Eq. S13 to obtain Eq. 8 can be considered as a shortcut in the derivation, it is the simplest way to ensure that Eq. 8 converges to Eq. S14 when k tends to zero, given the definition we used for discrimination ($^{13}\Delta$ and e). In practice, using our Eq. 8 or Eq. S13 adopted from Wingate et al. (2007) with the corresponding definitions for discrimination, has little impact on the modeling results (less than 0.5‰) and e values should be very comparable between the two studies.

For definition of symbols see main manuscript.

Methods S3 Modeling source water δ^{18}O

The oxygen isotope ratio of source water (R_s) was modeled based on a mass balance approach for the soil rooting zone (Ogée et al., 2009). The rooting zone water budget is expressed as:

$$\frac{dW_{soil}}{dt} = P - E_{tot} - D \quad (S16)$$

where W_{soil} (kg m^{-2}) is rooting zone water storage, and P, E_{tot} and D (kg m^{-2} s^{-1}) are precipitation, total evapotranspiration and drainage. We applied Eq. (S16) at a daily timescale to solve D, which was the only unknown as dW_{soil}/dt was derived from soil moisture measurements assuming a root zone depth of 0.2 m, E_{tot} was available from eddy covariance, and P was measured. R_s can then be solved from the budget of the rooting zone water ^{18}O:

$$\frac{dR_sW_{soil}}{dt} = R_{rain}P - R_sE_{tot} - R_sD \quad (S17)$$

where R_{rain} is the oxygen isotopic ratio of rainfall (measured on a monthly basis). Eq. (S17) assumes that all E_{tot} occurs without fractionation (cf. Ogée et al., 2009).

The resulting source water $\delta^{18}O$ composition was compared against the values observed for twig water suggesting 73% of the variation of twig water $\delta^{18}O$ was captured with the approach (Fig. S4). Generally measured twig water $\delta^{18}O$ lie within the range measured for soil water at depths 0.02 m and 0.1 m. During times when this was not true (e.g., late September 2019), modeled values typically also deviated from the measured twig water values. Plausible reasons for this are that root uptake is from deeper layers or that twig water is not in equilibrium with soil water due to low transpiration rates.

Methods S4 Derivation of Eqs. 10–12

When the sugar pool size is taken as a constant over time and $q = A_n$ (see section 3.2), the implicit solution of Eq. 6 (or Eq. 9 when neglecting e) is:

$$\frac{S_{sug}(R^t_{sug} - R^{t-1}_{sug})}{\Delta t} = (A_n + r_d)^t R^t_{assimilates} - (A_n + r_d)^t R^t_{sug} \tag{S18}$$

where Δt (s) is the time interval between $t-1$ and t. Solving for the isotopic ratio of sugars at time t yields:

$$R^t_{sug} = \frac{(A_n + r_d)^t R^t_{assimilates} + (S_{sug}/\Delta t) R^{t-1}_{sug}}{S_{sug}/\Delta t + (A_n + r_d)^t} \tag{S19}$$

Defining $\alpha = (A_n + r_d)/(S_{sug}/\Delta t + A_n + r_d)$ further simplifies the formulation to that presented in Eq. 10:

$$R^t_{sug} = \alpha^t R^t_{assimilates} + (1 - \alpha^t) R^{t-1}_{sug} \tag{S20}$$

Inserting the same formulation for the isotopic ratio of sugars at time $t-1$, $t-2$, ... into Eq. S20 yields:

$$R^t_{sug} = \alpha^t R^t_{assimilates} + (1 - \alpha^t)(\alpha^{t-1} R^{t-1}_{assimilates} + (1 - \alpha^{t-1})(\alpha^{t-2} R^{t-2}_{assimilates} + (1 - \alpha^{t-2})(...))) \tag{S21}$$

which can be written as a weighed mean of past time instances $R_{assimilates}$

$$R^t_{sug} = \sum_{n=0}^{\infty} (w_n R^{t-n}_{assimilates}) \tag{S22}$$

where w_n is the weight of the signal at time $t-n$:

$$w_n = \alpha^{t-n} \times \prod_{i=0}^{n-1} (1 - \alpha^{t-i}) \tag{S23}$$

To enable the calculation of weighted signals the sum in Eq. S22 was cut off at τ so that $\sum_{n=0}^{\tau} w_n \approx 0.95$. With this cut-off, the sum of the weights is less than unity unlike in Eq. S22 and therefore we need to add the sum to the denominator resulting in the formulation of Eq. 12:

$$R^t_{sug} = \sum_{n=0}^{\tau} (w_n R^{t-n}_{assimilates}) \Big/ \sum_{n=0}^{\tau} w_n \tag{S24}$$

References

Bernacchi, C. J., Singsaas, E. L., Pimentel, C., Jr, A. R. P. and Long, S. P. (2001) Improved temperature response functions for models of Rubisco-limited photosynthesis. *Plant, Cell & Environment*, **24**, 253–259.

Dewar, R., Mauranen, A., Mäkelä, A., Hölttä, T., Medlyn, B. and Vesala, T. (2018) New insights into the covariation of stomatal, mesophyll and hydraulic conductances from optimization models incorporating nonstomatal limitations to photosynthesis. *New Phytologist*, **217**, 571–585.

Farquhar, G. D., Caemmerer, S. V. and Berry, J. A. (1980) A biochemical model for photosynthetic CO_2 assimulation in leaves of C3 species. *Planta*, **149**, 78–90.

Farquhar, G. D., Ehleringer, J. R. and Hubick, K. T. (1989) Carbon isotope discrimination and photosynthesis. *Annual review of plant biology*, **40**, 503–537.

Farquhar, G. D., O'Leary, M. H. and Berry, J. A. (1982) On the relationship between carbon isotope discrimination and the intercellular carbon dioxide concentration in leaves. *Functional Plant Biology*, **9**, 121–137.

Kattge, J. and Knorr, W. (2007) Temperature acclimation in a biochemical model of photosynthesis: a reanalysis of data from 36 species. *Plant, Cell & Environment*, **30**, 1176–1190.

Kellomaki, S. and Wang, K.-Y. (1996) Photosynthetic responses to needle water potentials in Scots pine after a four-year exposure to elevated CO_2 and temperature. *Tree Physiology*, **16**, 765–772.

Launiainen, S., Katul, G. G., Lauren, A. and Kolari, P. (2015) Coupling boreal forest CO_2, H_2O and energy flows by a vertically structured forest canopy – Soil model with separate bryophyte layer. *Ecological Modelling*, **312**, 385–405.

Launiainen, S., Katul, G. G., Leppä, K., Kolari, P., Aslan, T., Grönholm, T., Korhonen, L., Mammarella, I. and Vesala, T. (2022) Does growing atmospheric CO_2 explain increasing carbon sink in a boreal coniferous forest? *Global Change Biology*, **28**, 2910–2929.

Medlyn, B. E., De Kauwe, M. G., Lin, Y.-S., Knauer, J., Duursma, R. A., Williams, C. A., Arneth, A., Clement, R., Isaac, P., Limousin, J.-M., Linderson, M.-L., Meir, P., Martin-StPaul, N. and Wingate, L. (2017) How do leaf and ecosystem measures of water-use efficiency compare? *New Phytologist*, **216**, 758–770.

Medlyn, B. E., Dreyer, E., Ellsworth, D., Forstreuter, M., Harley, P. C., Kirschbaum, M. U. F., Roux, X. L., Montpied, P., Strassemeyer, J., Walcroft, A., Wang, K. and Loustau, D. (2002) Temperature response of parameters of a biochemically based model of photosynthesis II. A review of experimental data. *Plant Cell and Environment*, **25**, 1167–1179.

Medlyn, B. E., Duursma, R. A., Eamus, D., Ellsworth, D. S., Prentice, I. C., Barton, C. V. M., Crous, K. Y., Angelis, P. D., Freeman, M. and Wingate, L. (2011) Reconciling the optimal and empirical approaches to modelling stomatal conductance. *Global Change Biology*, **17**, 2134–2144.

Mäkelä, A., Pulkkinen, M., Kolari, P., Lagergren, F., Berbigier, P., Lindroth, A., Loustau, D., Nikinmaa, E., Vesala, T. and Hari, P. (2008) Developing an empirical model of stand GPP with the LUE approach: analysis of eddy covariance data at five contrasting conifer sites in Europe. *Global Change Biology*, **14**, 92–108.

Ogée, J., Barbour, M. M., Wingate, L., Bert, D., Bosc, A., Stievenard, M., Lambrot, C., Pierre, M., Bariac, T., Loustau, D. and Dewar, R. C. (2009) A single-substrate model to interpret intra-annual stable isotope signals in tree-ring cellulose. *Plant, Cell & Environment*, **32**, 1071–1090.

Schiestl-Aalto, P., Stangl, Z. R., Tarvainen, L., Wallin, G., Marshall, J. and Mäkelä, A. (2021) Linking canopy-scale mesophyll conductance and phloem sugar $\delta^{13}C$ using empirical and modelling approaches. *New Phytologist*, **229**, 3141–3155.

Tarvainen, L., Wallin, G., Räntfors, M. and Uddling, J. (2013) Weak vertical canopy gradients of photosynthetic capacities and stomatal responses in a fertile Norway spruce stand. *Oecologia*, **173**, 1179–1189.

Wingate, L., Seibt, U., Moncrieff, J. B., Jarvis, P. G. and Lloyd, J. (2007) Variations in ^{13}C discrimination during CO_2 exchange by *Picea sitchensis* branches in the field. *Plant, Cell & Environment*, **30**, 600–616.

Yoshimura, K., Kanamitsu, M., Noone, D. and Oki, T. (2008) Historical isotope simulation using Reanalysis atmospheric data. *Journal of Geophysical Research: Atmospheres*, **113**, D19108.

Zhou, S., Duursma, R. A., Medlyn, B. E., Kelly, J. W. G. and Prentice, I. C. (2013) How should we model plant responses to drought? An analysis of stomatal and non-stomatal responses to water stress. *Agricultural and Forest Meteorology*, **182-183**, 204–214.
